# Burst of aneuploidy: a cost of adaptation driven by breakdown of cell cycle control

**DOI:** 10.1038/s44320-026-00217-6

**Published:** 2026-05-21

**Authors:** Adrian Pirog, Hanna Tutaj, Katarzyna Tomala, Ryszard Korona

**Affiliations:** Institute of Environmental Sciences, Faculty of Biology, Jagiellonian University, Krakow, Poland

**Keywords:** DNA Replication, Recombination & Repair, Evolution & Ecology

## Abstract

Complex genome rearrangements are intriguing because it remains unclear whether they occurred gradually or suddenly, and what promoted or impeded their development. We started by asking whether double loss of heterozygosity (dLOH) involving two different chromosomes is simply the result of two independent single LOH events. We examined hundreds of vegetatively growing diploid yeast strains with gene deletions known to stimulate LOH through different mechanisms. In dozens of these strains, dLOH mutants were overrepresented by tens or even hundreds of times. Interestingly, the (deleted) genes that caused the greatest instability were functionally diverse yet apparently affected the same biological process: cell cycle control. Furthermore, mutants were dominated by aneuploidy, which often involved multiple non-target chromosomes, with virtually no segmental loss, non-homologous recombination, or other small-scale changes. We conclude that the supply of separate, local rearrangements affecting only the required regions is insufficient, even in mutator strains. Consequently, double-pointed selection tends to reveal extensive reassortments resulting from systemic failures in mitotic cell division. Therefore, overcoming multiple adaptation barriers tends to produce aneuploidy, which is often reversible.

## Synopsis

This work demonstrates that double loss of heterozygosity in yeast arises mainly from transient bursts of genome instability linked to defective cell cycle control. These events drive widespread aneuploidy and may help illuminating mechanisms of complex genome evolution, including in cancer.

- A set of 392 diploid yeast deletion strains known, or suspected, to be prone to loss of heterozygosity (LOH) was used in intensive quantitative assays revealing the frequency of single (sLOH) and double (dLOH) events.
- Computational modelling has demonstrated that the observed frequencies of dLOH greatly exceed the expectations based on the independence of single LOH events, indicating frequent coincidental or burst-like genome destabilization.
- Excess dLOH is strongly associated with defects in cell cycle checkpoints and genome maintenance pathways, highlighting failures in cellular surveillance mechanisms.
- Genomic analyses showed that dLOH mutants are dominated by whole-chromosome aneuploidy rather than localized mutations, and that such instability is typically transient and selected against over time.

## Abstract (web summary)

Double loss of heterozygosity in yeast arises mainly from transient bursts of genome instability.

## Graphical abstract

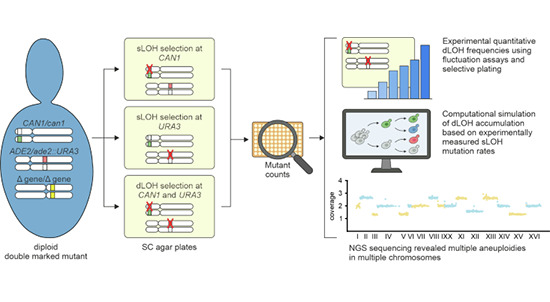

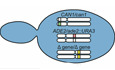

## Introduction

Asexual organisms and somatic cells replicate their genetic material and transmit it to daughter cells during mitosis. Considering a single gene in a diploid genome, the likelihood of its allelic composition (genotype) (Sunshine et al, 2015; Smukowski Heil, 2023)changing due to errors in mitosis is around a thousand times higher than due to replication errors resulting in a point mutation (Tutaj et al, 2022; Lang and Murray, 2008; Sui et al, 2020). This means that selection favoring a loss of function in a single gene will often promote the loss of a chromosome or a chromosomal segment containing this gene rather than mutations in the gene itself (Sunshine et al, 2015; Smukowski Heil, 2023). This could explain why aneuploidy is found in up to a quarter of new isolates of *Saccharomyces cerevisiae* (Zhu et al, 2016; Peter et al, 2018; Loegler et al, 2024). Nevertheless, what we see in nature is most likely only a small, biased sample of genome alterations, the origins and history of which are largely unknown. The current destabilizations could have developed in separate steps or all at once. They could originate as a response to new selective pressures or develop as a result of the initial destabilizations. They may be permanent or only temporary. The list of unknowns is long.

To overcome these difficulties, we developed a simple experimental system in which genome rearrangements were generated de novo. This enabled us to examine their frequency, the functional disturbances that trigger their formation, the architectural changes they involve, and their stability. Budding yeast is an ideal organism for such research given that methods for studying genome reassortments are well developed and intensely used, making it the best eukaryotic model in this area (Mulla et al, 2014; Segovia et al, 2015; Skoneczna et al, 2015). Our general approach was to detect incipient loss of heterozygosity (LOH) events in assays involving the loss of alleles that manifest themselves phenotypically. This simple yet efficient method enables a wide range of changes to be detected. These include the loss of an entire chromosome or part of it, segmental deletion following non-homologous end joining, replacement by a homologous chromosomal stretch via break-induced repair, and either local gene conversion or terminal reciprocal crossover after homologous recombination (Andersen et al, 2008). Collectively, these changes are indicative of genome stability issues that arise at various stages of the cell cycle (Siri et al, 2021; Huang and Koshland, 2003; Putnam et al, 2009).

The phenotypic assays of LOH have been applied extensively for large collections of mutant strains, greatly advancing our understanding of the molecular and cellular processes that are critical for genome stability. These were single LOH (sLOH) studies; that is, selection was concentrated at one site, and only what happened there was analyzed (Andersen et al, 2008; Hoffert and Strome, 2019; Strome et al, 2008; Huang et al, 2003; Hieter et al, 1985; Duffy et al, 2016; Yuen et al, 2007). It has been found that even one-site selection can result in rearrangements in other non-selected regions, and that, when selection is extended to two sites, complex rearrangements can become prevalent in otherwise normal diploid yeast (Heasley et al, 2020; Sampaio et al, 2020). This has led to the suggestion that rearrangements of genomes may often occur in the form of bursts of changes, which could play an important role in their evolution (Heasley et al, 2021). While this hypothesis appears plausible, it would be greatly advanced by identifying the functional aspects of such bursts, as this would help us to understand when and how frequently they occur, and what fitness consequences they typically entail. To this end, we have extended the two-point LOH selection to a broad collection of mutants known to affect genome stability due to the malfunctioning of defined components of cellular metabolism. We adopted the traditional genetic perspective that the malfunctioning of mutants can be used to deduce the workings of wild-type organisms (but also sought to verify this assumption, as discussed later).

In this experiment, LOH events were detected at one or both of two specific loci: *CAN1/can1* (at the end of the left arm of chromosome V) and *ADE2/ade2::URA3* (in the middle of the right arm of chromosome XV). We studied nearly 400 strains, each carrying a homozygous deletion of a single gene. This list was derived from previous studies that searched for deletions that increase (single) LOH, i.e., LOH mutators. We wanted to include possibly diverse LOH mutators, and therefore we compiled the results of experiments that employed two well-established but markedly different methods: one primarily designed to detect chromosomal rearrangements (gross chromosomal rearrangement, GCR) and the other to detect chromosomal losses (chromosome instability, CIN) (Stirling et al, 2011; Putnam et al, 2016). First, we performed extensively replicated assays at each of the two loci to obtain robust estimates of the sLOH rate per cell division. Using these estimates and simulations of mutation–accumulation, we tested whether the frequency of double LOH (dLOH) could be explained by two independent sLOH events. This enabled us to identify several dozen deletion strains in which dLOH was particularly prevalent. These strains were then studied further to determine which physical (structural) changes are most common in dLOH mutants and how stable the acquired alterations tend to be.

## Results

### The incidence of dLOH varies greatly between deletion strains

Regarding the aforementioned screens for gene deletions that decrease genome stability, the GCR assay identified 182 of them, while CIN identified 369. As 97 genes (ORFs) overlapped, 454 were unique. We intended to include all corresponding deletion strains in our strain construction and testing. However, this was not fully achievable as some ORFs had been disqualified from the YKO collection or were absent from our copy of it. Others were tried but excluded because we could not mutate them to *can1*, transform with *URA3*, or mate. The methods section details these manipulations, while Dataset EV1 lists both consecutive dropouts and the final set of 392 diploid strains obtained by us, each of which carries a different homozygous deletion (responsible for the mutator phenotype), as well as two heterozygous loci: *CAN1/can1* and *ADE2/ade2::URA3* (markers of destabilization).

**Figure EV1:**
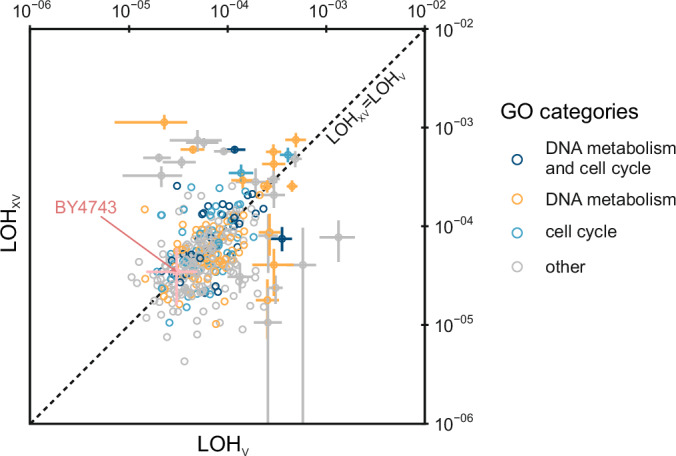
Relation between sLOH rates. Chromosome V (*CAN1/can1* locus) versus chromosome XV (*ADE2/ade2::URA3*) locus. Points represent the strains, *n* = 393, listed in Dataset EV2. Error bars refer to 95% confidence limits obtained through bootstrapping as described in Methods.

These strains contained deletions that had already been identified as causing greater instability, although some were rather less well-established than others. In this experiment, we obtained quantitative estimates of the mutation rate to sLOH by analyzing 20 replicate mutation–accumulation cultures of each of our newly constructed diploids at the *CAN1/can1* (chr V) and *ADE2/ade2::URA3* (chr XV) loci. These estimates are presented in Dataset EV2 and Fig. EV1. A significant proportion of the tested deletion strains—68% for *CAN1*, 82% for *URA3*, 42% for both*—*showed a higher rate of sLOH than the control strain at the two loci tested. We consider this to be a reassuring finding, given that we selected these strains based on qualitative results obtained in other laboratories, which were then confirmed in our quantitative assays.

**Figure 1:**
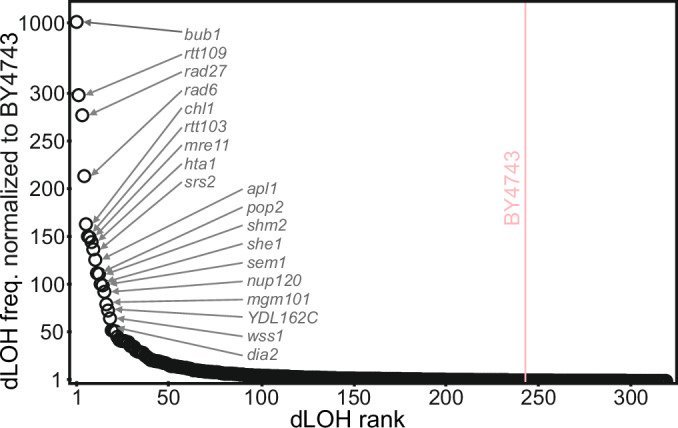
Frequency of dLOH in gene deletion strains. Ranked estimates of the average dLOH frequency. The pink vertical line shows the rank of the control strain BY4743. Source data are available online for this figure.

The mutation to dLOH does not need to arise as a single event affecting chromosomes V and XV; it could be a sequence of two mutations at two sites (see the next section of the Results). Therefore, rather than calculating the rate of dLOH mutation, we simply report the frequency of dLOH mutants as scored on agar containing both canavanine and 5-FOA. Dataset EV3 shows these estimates averaged over 20 replicate cultures. Figure 1 provides a graphical summary of these results and includes 314 deletions with non-zero average frequencies of dLOH, 242 of which were more unstable than the control strain. Deletion strains that did not produce visually detectable dLOH colonies were considered to be non-mutators and were excluded from further functional analyses. The strains exhibiting high instability under double selection were also more unstable under single selection regimes, as indicated by the correlation between the ranks of dLOH and sLOH averaged over two loci (Spearman’s *r* = 0.61; *n* = 314; *P* = 2.2e-16).

### Coincidental dLOH is much more frequent than expected from independent sLOHs

**Figure EV2:**
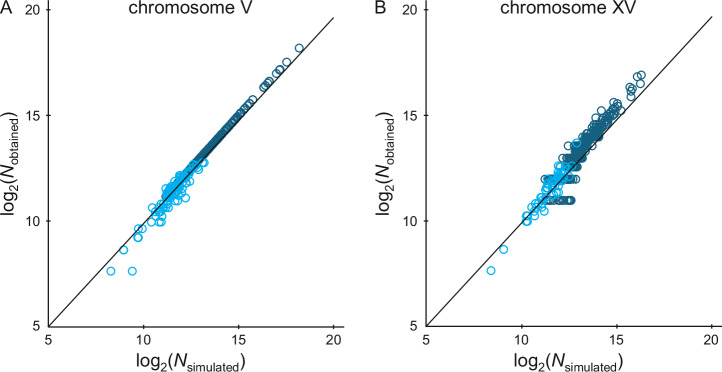
The correlation between the obtained and expected numbers of sLOH mutants. The correlation of sLOH mutants on chromosomes V (**A**) and XV (**B**) is shown across the set of studied deletion strains. The mutation rate *μ* of sLOH was calculated from the “obtained” (empirical) counts as described in the “Methods” section. These *μ* estimates were then used to simulate the mutational process in replicate cultures to obtain a simulated number of mutants. The numbers shown in the graphs are the median (dark) or mean (light) counts in the replicate cultures. The latter were used for cases with a low number of mutants, with the median being zero

It was not possible to distinguish experimentally between a one-step or two-step origin of dLOH. Therefore, we sought to achieve this through computer simulations using the empirically estimated rates of single LOHs. These were precise, with their 95% confidence limits typically falling within a range of 0.75–1.25 of the central tendency (see Dataset EV2). To test for unbiasedness, we first simulated the accumulation of sLOH mutants only, finding that the calculated and empirically obtained numbers were in good agreement (see Fig. EV2). Reassured by this result, we ran simulations mimicking the accumulation of dLOH mutants. During cell division, one of the progeny cells could become a sLOH mutant at its respective rate, or a ‘coincidental’ dLOH mutant at a rate equal to the product of the two individual sLOH rates. The sLOH mutants could then acquire the other mutation, turning into a “sequential” dLOH mutant. Results of these simulations are listed in Dataset EV4. An important caveat here is that, although we obtained empirical estimates of the strain-specific sLOH rates, we were unable to derive comparable estimates for the fitness effects of sLOH and dLOH mutants, as these effects were dynamic and effectively undetectable in the multiple mutation–accumulation cultures. We therefore performed simulations in which the fitness costs of sLOH and dLOH were set arbitrarily at 10% and 20%, respectively. As expected, the number of simulated dLOH events decreased considerably, thereby making the excess of empirical dLOH scores even more pronounced (see Dataset EV5). However, our primary objective was to demonstrate that empirical dLOH frequencies are higher than expected. To be conservative in our modeling, we set all fitness costs at zero to demonstrate that even unrestricted propagation of LOH mutants in simulated colony growth does not produce dLOH mutants in amounts comparable with those observed empirically.

**Figure 2:**
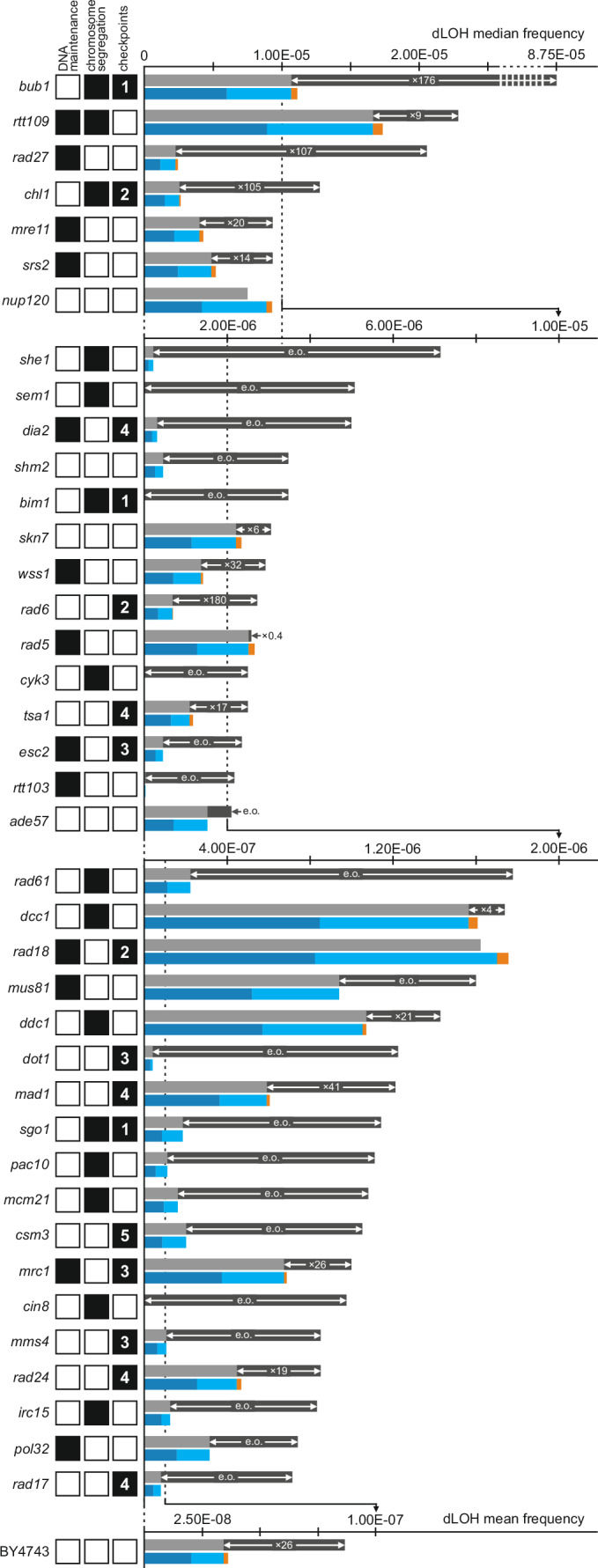
Functional profiles and mutant frequencies of the top 39 dLOH mutator strains. Right panel: For each deletion strain, the upper gray bar shows the empirically measured median frequency of dLOH, while the lower bar shows the result of a simulation in which dLOH occurred either sequentially (dark blue: first loss of *CAN1*, then *URA3*; light blue: first loss of *URA3*, then *CAN1*) or simultaneously at two loci (orange). White arrows indicate the proportion of empirically determined dLOH frequencies that cannot be explained by the sum of the sequential scenarios, and the white numbers specify how many times this exceeds the predicted frequency of simultaneous dLOH origin (see Dataset EV5). Strains for which such a comparison was not possible due to the lack of data for dLOH obtained from simulation are marked as e.o. (experimental only). Scale bar: Note that the scale bar of mutant frequency is broken into four pieces to accommodate the wide frequency variation. In the case of BY4743, the mean instead of the median frequency is used. Left panel: Annotations of Gene Ontology (GO) categories related to DNA maintenance (mostly repair), chromosome segregation (mostly sister chromatid interaction or spindle formation), or a cell cycle checkpoint: (1) mitotic spindle assembly/orientation checkpoint, (2) mitotic G1 DNA damage checkpoint, (3) mitotic intra-S DNA damage checkpoint, (4) mitotic DNA damage/integrity checkpoint, (5) DNA replication checkpoint (see Dataset EV6 for details of GO analysis). Source data are available online for this figure.

Figure 2 compares the observed and expected frequencies of dLOH for the 39 most unstable deletion strains. These strains were selected based on their dLOH scores, but their sLOH estimates also tended to be among the highest, as reported above. The first 19 strains form a clearly distinct group, as do the remaining 20 strains, although probably to a lesser degree (see Fig. 1). The frequency of dLOH obtained through experimentation was almost always higher than that predicted by simulations. The overall frequencies of dLOH observed empirically and presented in Fig. 2 are divided into two components: one that can be attributed to the sequence of two independent sLOH (light gray bar), as suggested by simulations (light and dark blue bars); and another that cannot be explained in this way (dark gray bar) and is therefore considered coincidental. The latter component was then compared with the predicted frequency of coincidental dLOH formation, calculated as the product of two sLOH rates (orange bar). The empirically observed frequencies exceed the predicted frequencies by tens or even hundreds of times. This is shown as numbers within the bars on the right-hand side of the graph (see also Dataset EV5). We attribute the excess dLOH mutants to “coincidental” events. These could be short periods of hyper-instability or one-step events. The latter appeared to be a suitable suggestion, as the data from DNA sequence analysis revealed, and this will be discussed later.

A previous study reported that the product of the two sLOH rates was 14–150 times lower than the dLOH rate estimated from the overall count of dLOH mutants identified in two different wild-type diploid yeast strains (Sampaio et al, 2020). However, as mentioned above, estimates of the dLOH rate obtained in this way are necessarily inflated, as they account for sequential dLOH events, rather than coincidental ones only (as suggested by using the product of the sLOH rates as a benchmark). Here, we were able to subtract the predicted sequential dLOH events from the total observed, and demonstrate that the remaining events were 26 times more frequent than could be predicted from two independent, yet coincidental, sLOH events (i.e., the product of the sLOH rates; see bottom of Fig. 2). Thus, our results are consistent with earlier reports and, taken together, they demonstrate that complex genome changes, as exemplified by dLOH, appear to comprise linked rather than distinct individual rearrangements in both mutator and wild-type strains. The advantage of using mutators is that they help us to understand the functional basis of this phenomenon, as detailed below.

### Excessive dLOH is linked to failures in cell cycle control

We searched the Saccharomyces Genome Database (SGD) for the Gene Ontology (GO) categories enriched among the deleted genes in the most unstable strains. A summary of these analyses is presented in Fig. 2 (three columns on the left), where additional details are provided. Almost all of the genes are related to either DNA maintenance (mostly repair) or chromosome behavior (e.g., sister chromatid interaction and spindle engineering). The third column highlights genes that are involved in checkpoints of cell cycle progression over its several stages. These genes account for nearly half of the list, even though the term encompassing all these checkpoints, GO:0000075 “cell cycle checkpoint”, is annotated to only 1.3% of all yeast ORFs. Restricting analyses to the genes/ORFs that were included in the present work, we still find that the cell cycle checkpoint category is significantly overrepresented among those with the highest dLOH frequency (Dataset EV6). The remaining genes, which lack an explicit “checkpoint” annotation, are often those that are particularly important for DNA, chromatid, or spindle integrity. This means that strains lacking these genes may sustain such extensive damage that even undisturbed checkpoints are ineffective. All of this strongly suggests that genomes are susceptible to complex rearrangements when checkpoints for DNA or spindle integrity fail to stop the progression of the cell cycle.

### DNA content in incipient dLOH mutants is often substantially increased or decreased

**Figure 3:**
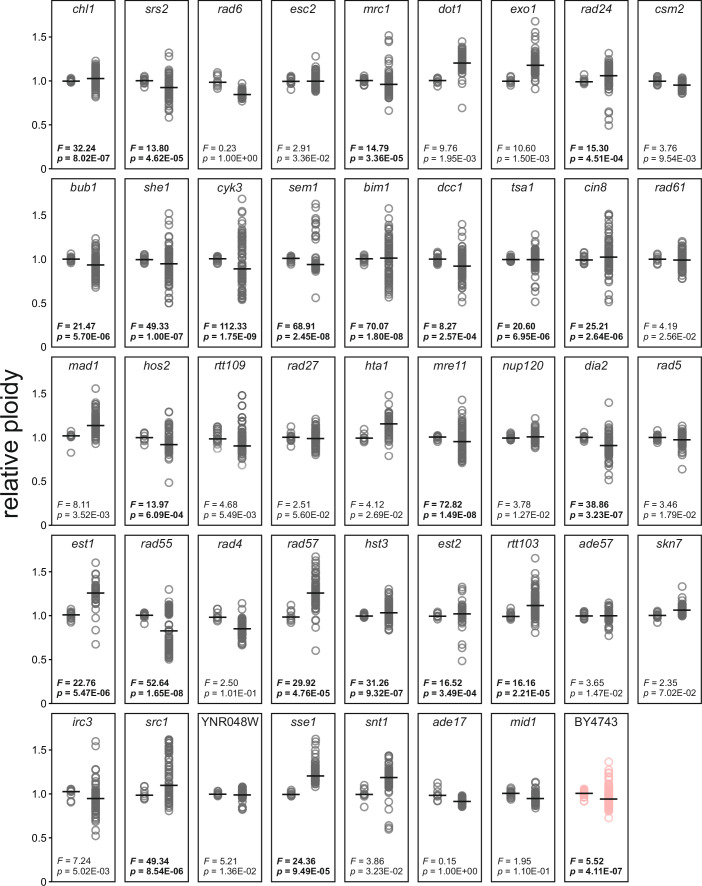
Change in DNA content in dLOH mutants. Flow cytometry estimates of DNA content in replicate assays of an ancestral deletion strain (left side within each pair) compared to that of a sample of dLOH mutants (right). DNA content of the mutants is normalized to the median of the ancestral estimates. The horizontal bars represent the median for each strain. The ratio of mutant to ancestral variances are shown as the *F* statistics in the Fisher’s test for variance homogeneity, the associated *P* values that are significant at the 0.05 level when accounting for multiple comparisons (after Bonferroni’s correction) are shown in bold. Source data are available online for this figure.

We measured the cellular content of DNA using flow cytometry in 43 deletion strains with the highest frequency of dLOH. These were essentially the most unstable strains from Fig. 2, with a few removed or added to make the sample more functionally diverse. For each strain, we examined approximately 80 independent original colonies of the dLOH mutants and compared them with replicate colonies of their non-mutated ancestors. Figure 3 shows that, in many deletion strains, the dLOH mutants generally exhibited greater variation in DNA content than their ancestral strain. To quantify this increase, we calculated the ratio of the variance in DNA content among the dLOH mutants to the variance among the ancestral replicates (the strain-specific *F*-statistic). As indicated in the figure, we found that the increase in variation was often statistically significant. We also tested whether the extent of DNA gains or losses (as measured by *F*) correlated with the frequency of dLOH. However, no such association was observed (Spearman’s *r* = 0.162, *n* = 44, *P* = 0.255). This could mean that aneuploidization did not occur in all of the most unstable strains, and that others may have acquired different changes to their genomes, such as point mutations. Therefore, detailed DNA sequence analyses appeared necessary; we report them below.

### Whole-chromosome aneuploidy dominates in dLOH mutants

**Figure EV3:**
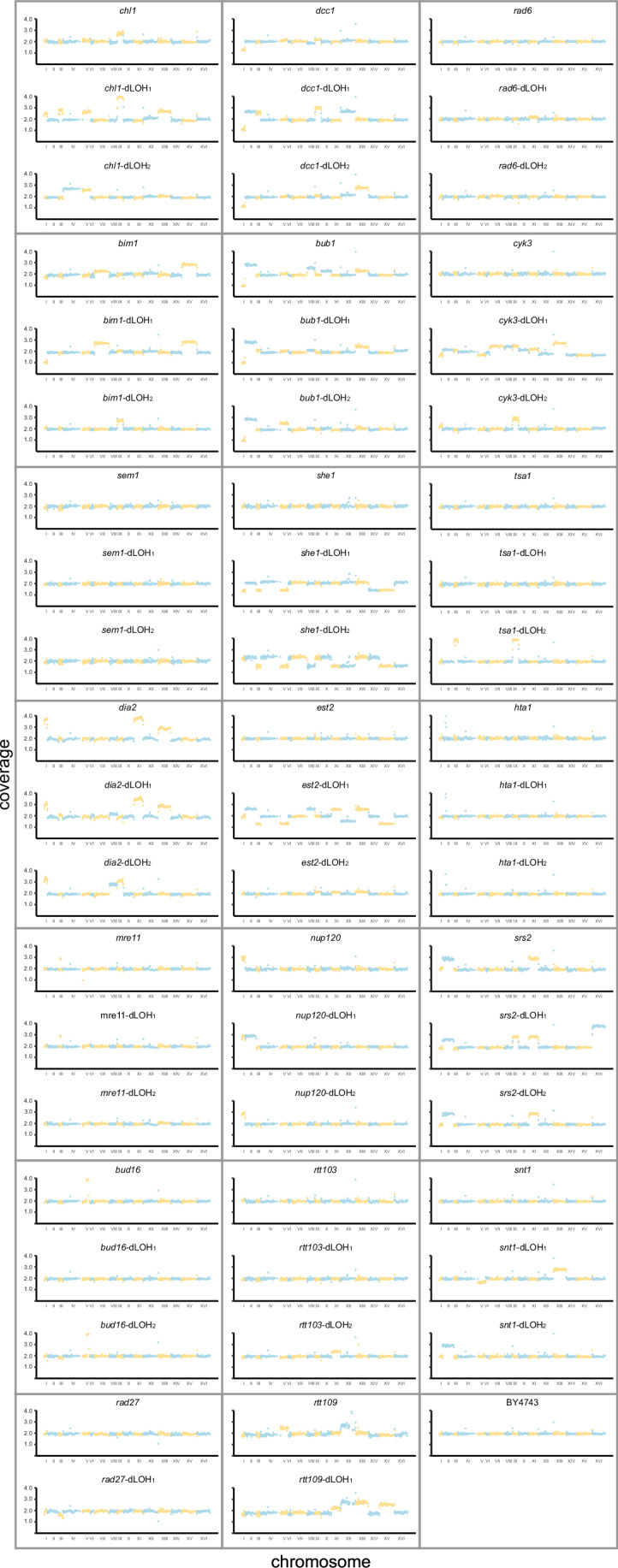
Coverage of DNA sequencing. Plots show the sum of DNA reads per 20 kb chromosome fragment, normalized to diploidy (or triploidy for *she1*).

We further investigated a sample of 20 deletion strains by performing next-generation DNA sequencing (NGS). Those strains were selected from among those shown in Fig. 2 and were chosen to represent both minimal and large changes in DNA content, as well as a range of distinct biological processes. The aim was to compare the ancestral strains with two dLOH mutants derived from them. The latter were selected to represent increases and decreases in cellular DNA content, as detected by flow cytometry, if present. A simple analysis of coverage by DNA reads suggested that most chromosomes were essentially diploid. However, chromosomes other than 2*n* were often not strictly mono- or trisomic, but “non-integer”. Importantly, these non-integer scores remained consistent throughout the entire chromosome, indicating that the colonies contained clones with different numbers of particular chromosomes (see Fig. EV3).

**Figure 4:**
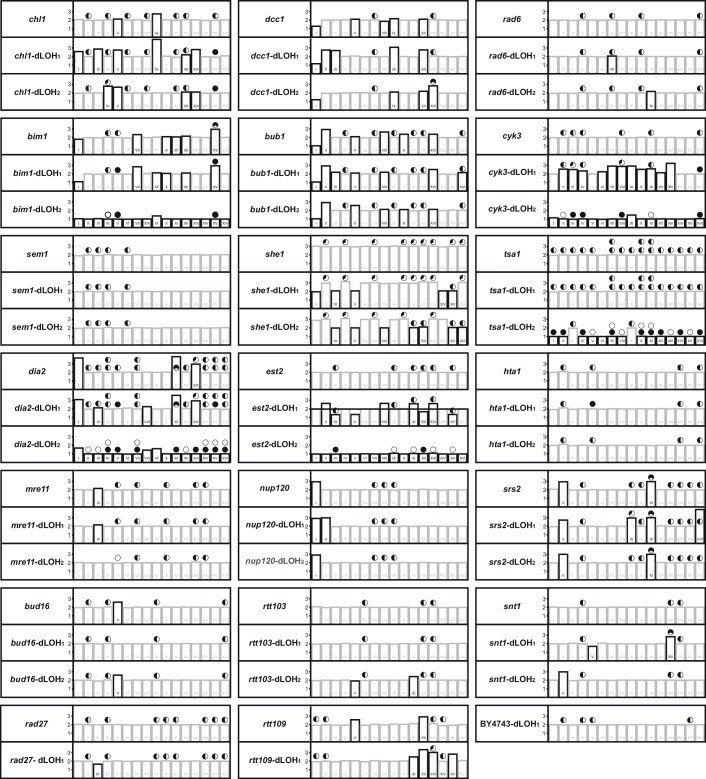
Individual chromosome ploidy in dLOH mutants. Next-generation DNA sequencing data were used to determine the ploidy level of individual chromosomes of an ancestral strain (top of each set) and two mutant strains (middle and bottom). Thick lines indicate chromosomes whose ploidy level differs from the ancestral level, as revealed by ANOVA with Tukey’s post hoc test. Ancestral ploidy was inferred from data on spontaneously arising polymorphic variants (split circles), which either persisted or disappeared in mutants (full or empty circles). All ancestral strains were essentially diploid, as expected, except for the triploid *she2*. Source data are available online for this figure.

Figure 4 shows the results of the quantitative analysis of departures from diploidy. For each ancestral or dLOH strain, we summed the DNA sequence reads per chromosome, divided the scores by the chromosome length, and compared the resulting 16 average coverage estimates using a one-way ANOVA, followed by Tukey’s post hoc comparisons. In nearly all strains, there were chromosomes that deviated from the modal coverage value. To determine whether these modal values were indeed the assumed 2*n*, we used the signals obtained in the cytometric assays together with the heterozygosity information obtained by NGS. To this end, we examined heterozygous positions, substitutions, and small insertions/deletions (indels) that were present in the ancestral strains but could disappear in the dLOH mutants (Datasets EV7 and EV8). For example, *dia2* had heterozygous positions, one or two, in several chromosomes in the ancestral strain (presented as black-white half-circles). In one dLOH mutant, the heterozygosities were still present, indicating the strain remained diploid. The other dLOH mutant tended to have only one allele (presented as black or white circles), indicating haploidy as the modal amount of DNA. Similar reasoning was applied to all strains.

A close examination of Fig. 4 reveals that aneuploidy occurred in almost every dLOH mutant, although this often partially regressed towards 2*n* status. In total, there were 205 cases when summed over all chromosomes in all strains depicted in the graphs. This equates to an average of 12.8 cases per chromosome. Paradoxically, the chromosomes targeted by selection, V and XV, had scores close to the average: 20 and 11, respectively. Overall, the picture appears clear: selection for two loci, each on a different chromosome, reveals general destabilization events rather than those centered on the target sites.

### Selection for dLOH mutants often results in bursts of out-of-target aneuploidy

Although the general destabilization conjecture is strongly supported by statistical evidence, we undertook direct genetic analysis to confirm it. We selected four deletion strains in which the excess of dLOH was particularly evident: *sem1, chl1, mre11*, and *rad27* (see Fig. 2). We collected a number of dLOH mutants for each strain using the canavanine plus 5-FOA selective medium. These mutants were then tested for the retention of heterozygosity at the *LYS2/lys2* and *MET15/met15* loci, which reside on chromosomes II and XII, respectively (standard in all BY diploid strains). Of the 61 dLOH mutants for *sem1*, 16 lost heterozygosity on chromosome II, 14 on chromosome XII, and 7 on both II and XII. For *chl1*, the numbers were: 108, 4, 8, and 1; 120, 13, 9, and 6 for *mre11*; 144, 4, 16, and 0 for *rad27*. Thus, the frequency of additional LOHs in the dLOH strains was extraordinarily high, given that LOH on chromosome II is normally as rare as on chromosomes V and XV, that is, one to a few per ten thousand cells. LOH on chromosome XII is normally about ten times more frequent, yet still far rarer than observed here (Tutaj et al, 2022).

Cross-validation of statistical analyses based on DNA counts (Fig. 4) and additional experimental data (reported above) strongly supports the idea that a large proportion of dLOH mutants arose coincidentally or nearly coincidentally in short periods of instability. The ‘sequential’ model could be realized if the fitness cost of the initial sLOH event was either absent (e.g., due to crossover or gene conversion) or offset (e.g., due to endoduplication of a monosomic chromosome). This would allow sufficient time for a second sLOH event to occur. Even in this ‘permissive’ scenario, there is a striking overrepresentation of dLOH (see Fig. 2). In DNA data, we rarely observe one or two changes only at the V and XV chromosomes, that is, the chromosomes targeted by selection, while no alterations are present on the other chromosomes. In summary, the relatively frequent and extensive aneuploidization extending beyond the selection targets is well confirmed.

### Point mutations and segmental losses, gains, or rearrangements are very rare

There were a few changes other than those affecting whole chromosomes. The heterozygous point mutations mentioned above were rare in the ancestral deletion strains, ranging from a few (*dcc1, rad6, bim1, sem1, hta1, nup120, rtt109, bud16, rtt103, snt1*) to over 20 (*tsa1, dia2*) per whole genome. Even rarer were heterozygous mutations present only in the dLOH mutants, which had to arise between their construction and their use in the LOH screens. This was not due to a lack of sensitivity; we detected all the changes that we intentionally introduced: the spontaneous point mutations within the *CAN1* locus (Dataset EV7). We also looked specifically for amplified fragments composed of two non-adjacent sequences, as this could indicate non-homologous recombination leading to inversion or translocation. No convincing events of this type were found. Again, we were able to see all the hybrid fragments we expected: the recombination sites at the borders of the *ADE2* locus (Dataset EV8). Having established that substitutions, short indels, and recombination sites are so rare, we then asked whether copy number variation involving DNA segments of any length was present. Two independent analyses looking for DNA segments enriched relative to the 2*n* baseline showed that this was not the case. The results are reported and graphically summarized in Dataset EV9. With the exception of whole chromosome gains and losses, there were essentially no copy number variants in non-telomeric regions that would be detected by both analyses, which we considered a criterion of reliability. Together, these findings suggest that large-scale aneuploidy is the dominant feature of the mutational landscape of dLOH mutants, with smaller-scale sequence alterations remaining relatively rare.

### Experimental evolution of dLOH mutants leads to a decline in aneuploidy

**Figure EV4:**
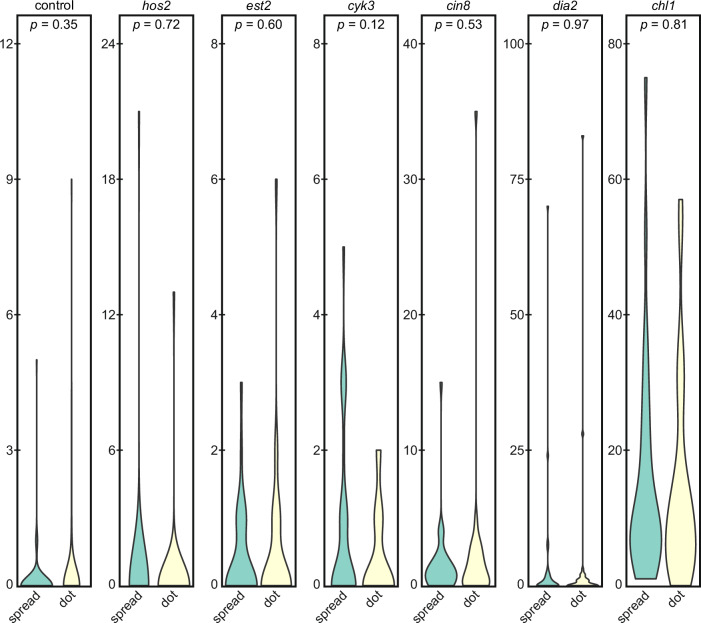
The number of dLOH mutants in miniaturized and standard plate assays. Samples were taken from the same replicate cultures in parallel and exposed to canavanine plus 5-FOA, either as whole-plate spreads or as dots that mimicked the conditions used in the miniaturized assay. The *P* values were obtained in a Wilcoxon’s test comparing the scores obtained in the two assays for each gene separately.

As shown in Figs. EV2 and  4, some chromosomes were not haploid, diploid, or triploid. This suggests that we were isolating DNA from a mixture of strains (genetically heterogeneous populations). It is highly unlikely that our original dLOH colonies were derived from more than one mutant cell. Heterogeneity probably developed during colony growth and then during the preparation of the liquid culture for DNA isolation. This is because the loss of an extra chromosome in trisomic variants or non-disjunction in monosomic variants results in a higher growth rate, leading to the spread of re-diploidized variants.

In order to test the plausibility of this scenario and its potential for continued existence, we thawed the liquid cultures used in the cytometric assays and cultivated them in batch cultures for 200 generations. These evolved populations were analyzed in the same way as the original cultures, enabling us to compare the DNA content of the cells before and after this experimental evolution. This was done for 21 original deletion strains, which generally had the highest frequency of dLOH mutants. Trios of mutants were selected for each deletion strain. The aim was to achieve a wide range of departures from diploidy, but three dLOH mutants with the highest and three with the lowest cytometry scores were excluded as they were potentially unrepresentative. We then identified three groups of five mutants with the lowest, medium, and highest DNA content, randomly selecting one mutant from each group.

**Figure 5:**
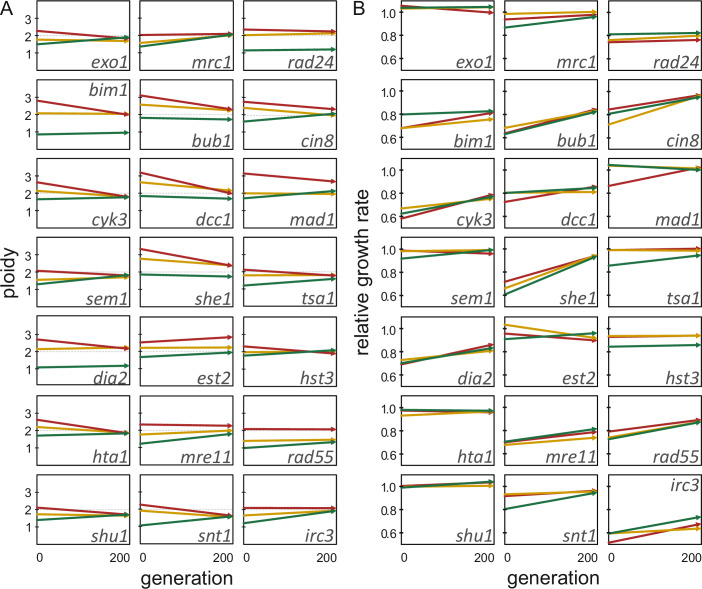
Experimental evolution of dLOH mutants. (**A**) Each arrow represents a change in ploidy level after 200 generations of batch culture. Mean values of three independent replicates of a strain with high (red), medium (yellow), and low (green) initial DNA content are shown. ”Ploidy” is the amount of DNA divided by that of the wild-type BY4743, with two meaning diploidy. (**B**) Changes in the maximum growth rate of the same strains; the growth rate is related to that of BY4743. Source data are available online for this figure.

Figure 5A shows a virtually uniform pattern of coalescence towards diploidy across all strains. Changes in the cellular content of DNA were least significant when initially close to 2*n*, and most significant when initially higher or lower. We also measured the growth rate of the isolates in the exponential phase, both before and after evolution. Figure 5B shows that most of the evolved isolates grew faster, with the greatest relative improvement seen in strains that diverged the most from 2*n*. However, this does not necessarily mean that true diploidy would be achieved if evolution were to continue for much longer. Some aneuploidies may have been beneficial in the specific propagation regime, while others may have compensated for the disturbances introduced by the gene deletions studied, which are often substantial (SGD). However, it is evident that selection drives aneuploidy to contract rather than stabilize or develop through a runaway process.

## Discussion

We developed an experimental system to study complex genomic changes from two complementary perspectives: their origin and their evolutionary consequences. Our model of complex rearrangement was based on an event of double LOH. We investigated how frequently this occurs and which defects in cellular metabolism promote it. These defects were introduced through mutant strains that are known to increase the likelihood of chromosome rearrangement or destabilization. Regarding the rate and dynamics of dLOH origin, we considered the possibility that they developed through the independent occurrence of single LOHs. This would result in mostly sequential and very rarely coincidental dLOH ascendance. However, our simulations showed that even if there were no associated fitness costs, which is unrealistic, the emergence of dLOH could not be explained as a combination of independent sLOH events, including both sequential and coincidental ones. Therefore, dLOH mutants must arise relatively frequently, depending on each other or on some other underlying cellular events.

Two kinds of evidence have revealed the molecular and cellular basis of the numerical excess of dLOH: functional annotation analyses of the most unstable deletion strains, and DNA sequence analyses of the dLOH mutants that arose in them. The former indicated that dLOH was particularly prevalent in strains in which the cell cycle progressed despite DNA or spindle damage not being addressed. The second confirmed this by revealing the extent of genome alterations, which were not limited to the two sites targeted by selection, but were much broader, as if the general program for orderly chromosome replication and segregation had been disrupted. Indeed, when we collected and tested additional dLOH mutants for LOH on two more chromosomes, we found triple or even quadruple LOH events at an extraordinarily high frequency.

The high prevalence of extensive changes, rather than those narrowly focused on regions containing target genes, suggests that the supply of alterations limited to adaptive needs is insufficient, even in mutator strains. In wild-type strains, single events of LOH occur at a rate of about one in ten thousand cell divisions, so a fortuitous combination of two of these events must be exceedingly rare (Tutaj et al, 2022). Therefore, the required double alteration in a wild-type strain is most likely to be achieved when a broad change involving the relevant points occurs (Sampaio et al, 2020). Here, we used hundreds of deletions that increased LOH by altering various aspects of cellular metabolism, with the aim of increasing the frequency of local genome alterations. Many of these deletions have been specifically linked to the occurrence of such rearrangements (Putnam et al, 2016). However, we found that dLOH events involving multiple entire chromosomes were especially prominent. Apparently, extensive aneuploidization was sufficiently prevalent to mask events involving combinations of more localized genomic changes.

Although we used mutants with a permanent and complete absence of certain gene functions, our aim was to gain broader (universal) insights into the general mechanisms underlying complex genome destabilization. Even normal, genetically stable cells do not function in a single, unchanging mode. A recent study has shown that wild-type strains undergo periods of phenotypic instability, with double mutation rates reaching up to 17 times the level expected based on experimentally determined single mutation rates (Agier et al, 2025). Although that study involved lesions of a smaller scale, such as point mutations, segmental duplications, and reciprocal translocations, aneuploidies are also likely to result from the transient malfunctioning of individual genes or gene networks. Our data show that among the various possible metabolic errors, those that risk disrupting cell cycle control are the most consequential for complex genome changes. This conclusion likely applies regardless of whether the errors are genetic or purely phenotypic in origin.

Our goal was not only to elucidate the molecular and cellular mechanisms underlying the emergence of complex genome changes, but also to understand their potential role in evolution. The term ‘burst’ appears to be particularly well-suited to the evolutionary dynamics of this phenomenon, as such alterations appear to be inherently transient, bringing about a substantial decrease in fitness and prompting natural selection to dampen or eliminate them. The speed at which selection operates in the original colonies makes it virtually impossible to capture the full spectrum of incipient mutants, even under laboratory conditions that stabilize the environment and enable changes to be monitored. We believe that our experimental model illustrates what happens in yeast evolution in the wild, in industrial facilities, and in patients, though these events are far more challenging to observe there directly. In general, selection tends to conceal evolutionary history, especially if past events were associated with high fitness costs. Therefore, finding an original genomic ‘hopeful monster’ would be intrinsically difficult if only its least unpalatable characteristics were permitted to persist (Theißen, 2006).

Paradoxically, our findings may be particularly relevant to cells other than yeast cells. In free-living organisms, a major component of fitness is growth rate, which typically declines under aneuploidy. Extensive genomic changes potentially become helpful only in a rather special situation, such as exposure to multiple toxins. The evolution of somatic cells in multicellular organisms is likely less restricted in this respect. They are programmed not to divide unless specifically permitted to do so. Therefore, there is no immediate cost to genome restructuring in terms of slowed growth, but instead an opportunity for the brakes on cell division to be loosened incidentally. One of the best-known examples of this process is the loss of a wild-type allele of a tumor suppressor gene, known as the “second hit”, which reveals a pre-existing recessive mutation (Knudson, 1971, 2001). Other examples of mechanisms hiding genetic variants that can be exposed by genome rearrangements include non-allelic compensation and balanced copy number variation (Ryland et al, 2015). Our study indicates that disruptions to cell cycle control can be an important source of chromosomal rearrangements, facilitating the breakdown of multiple barriers in somatic evolution, such as those observed in cancer.

## Methods

**Table:** Reagents and tools table

Reagent/resource	Reference or source	Identifier or catalog number
**Experimental models**		
*Saccharomyces cerevisiae* (Yeast Knockout Collection (YKO)	Baker Brachmann et al, 1998	BY4741, BY4742
**Recombinant DNA**		
Not applicable		
**Antibodies**		
Not applicable		
**Oligonucleotides and other sequence-based reagents**		
PCR primer 1F	Genomed	ATCGGACAAAACAATCAAGTATGTCGAAAGCTACATATAA
PCR primer 1R	Genomed	TATATCAATAAACTTATATATTAGTTTTGCTGGCCGCATC
PCR primer 2F	Genomed	AAGTGATTGACTCTTGCTGA
PCR primer 2R	Genomed	CCTTGCTTCTTGTTACTGGA
**Chemicals, enzymes, and other reagents**		
CSM—Arg-Ura	Formedium	DCS1199
Yeast Nitrogen Base w/o aa	Sigma	Y0626-250G
D-glucose anhydrous	Bioshop	GLU501.1
Agar	BTL	S-0030
Uracil	Bioshop	URA241.100
Adenine sulfate	Bioshop	ADS201.50
Arginine	Sigma-Aldrich	A5131-100G
Lysine monohydrate	Sigma	L5626-100G
Glycerol	Bioshop	GLY002.1
L-Canavanine	Sigma	C9758-5G
5-Fluoroorotic Acid	Formedium	5FOA10
PEG 3500 50% w/v	Bioshop	PEG335.1
LiAc 1.0 M	Acros Organics	199840010
ssDNA	Bioshop	DNA002.1
DNA polymerase Taq	Thermo Scientific	#EP0404
PCR buffer	Thermo Scientific	#EP0404
dNTPs	Thermo Scientific	#R0241
Propidium iodide	Sigma-Aldrich	P4864-10ML
RNAza A	A&A Biotechnology	1006-50
Genomic Mini AX Yeast	A&A Biotechnology	058-60
**Software**		
Gene Ontology Slim Term Mapper	https://www.yeastgenome.org/goSlimMapper	
RStudio (4.2.3)	https://www.r-project.org/	
ggplot2		
BWA-MEM (v0.717-r1188)	Li and Durbin, 2009	
Samtools (v1.15.1)	Danecek et al, 2021	
MarkDuplicates (Picard Toolkit 2019 v2.27.1)	https://gatk.broadinstitute.org/hc/en-us/articles/360037052812-MarkDuplicates-Picard	
GATK 4.4.0		
Mutect2 software from GATK (Genome Analysis Toolkit) (v4.2.6.1)		
CNVpytor v1.2		
CNVkit v0.9.10		
VEP tool (v104)		
smoove (v0.2.6)	https://github.com/brentp/smoove	
LUMPY	Layer et al, 2014	
bedtools intersect (v2.30.0)		
OpenAI, GPT-4	https://openai.com/contributions/gpt-4v/	
**Other**		
pRS416 plasmid	ATCC	#87521
*S. cerevisiae* R64-1-1 supplemented with the *kan*MX4	Ensembl (v104)	
*S. cerevisiae R64-1-1 GTF*	Ensembl (v104)	
epMotion® 96	Eppendorf	5069000110
Tecan Infinite® M200	Tecan	30018573
CytoFLEX	Beckman Coulter	B4-R0-V0 PN: B50960 AA
Illumina Novaseq PE150	Illumina	

### Initial strains, growth conditions, and media

**Figure 6:**
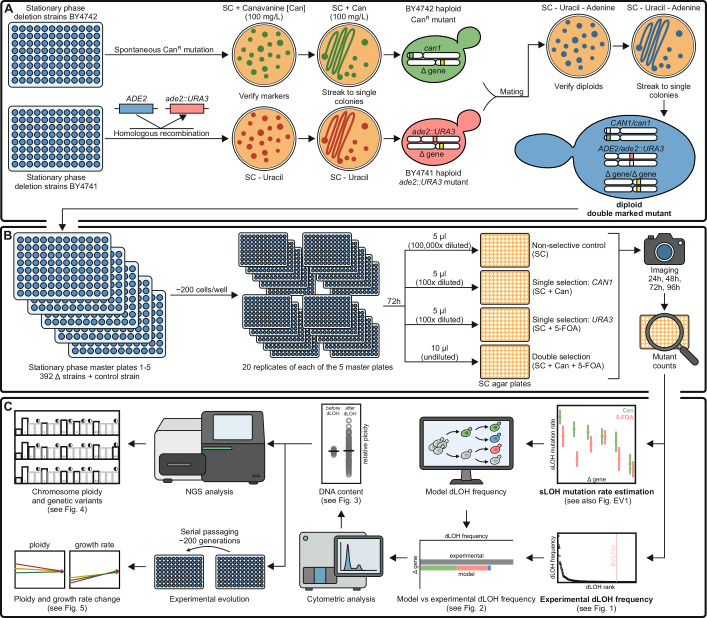
Overview of experimental procedures. Upper panel (**A**): derivation of diploid test strains, each with a unique single-gene homozygous deletion accompanied by the same two heterozygous counter-selectable loci used to detect LOH (loss of heterozygosity). Middle panel (**B**): the above test strains were replicated into independent mutation–accumulation cultures, which were appropriately diluted and plated onto non-selective, single-selective, and double-selective agars to obtain countable numbers of colonies. Lower panel (**C**): the empirical counts were used to calculate single LOH rates, after which computer simulations were applied to compare the predicted and obtained frequencies of double LOH mutants; samples of dLOH mutants were then subjected to cytometric assays of DNA level, DNA sequence analysis, and experimental evolution.

A schematic description of the experiments is provided in Fig. 6A–C. We used strains from the Yeast Knockout Collection, YKO (Baker Brachmann et al, 1998). These were haploid strains BY4741 *MAT***a** *his3 leu2 ura3 LYS2 met15* and BY4742 *MATα his3 leu2 ura3 lys2 MET15*, each carrying a single deletion of a non-essential gene. Of the 454 unique genes selected for this study (see the first section of “Results”), our collection of deletions contained 447 of the BY4741 *MAT***a** strains and 444 of the BY4742 *MATα* strains. All the accessible strains were arrayed on 8-by-12 format titer plates with flat-bottomed wells and stored in a deep freezer. They were then used to inoculate all subsequent liquid cultures with a 96-pin pipette. The same pipette was used to deposit small samples of cultures (up to 10 µL) on agar-solidified media. Standard synthetic complete (SC) medium, deprived of or supplemented with appropriate ingredients, was used in all growth and selection experiments. Cultures were of 200 µL volume, incubated at 30 °C without agitation but thoroughly mixed before being sampled. The selected original haploids and all strains derived from them were stored at −70 °C with 25% glycerol added.

### Construction of strains

The BY4742 *MATα* deletion strains were grown to the stationary phase in 200 µL aliquots and then overlaid on SC agar lacking arginine and supplemented with 100 mg/L canavanine. Most strains yielded multiple Can^R^ mutants on the first try. Unproductive attempts were repeated until up to 1 mL of stationary phase culture was used. The procedure was successful for 414 deletion strains (Dataset EV1). One mutant per deletion strain was randomly selected, streaked twice to single cells, and stored. The wild-type BY4742 (with no deletions except for the trophic markers listed above) was subjected to the same protocol to obtain a control Can^R^ strain.

In the case of the opposite mating type, BY4741 *MAT***a**, we started with a wild-type strain and replaced its *ADE2* locus with *URA3* amplified from plasmid pRS416 using primers 1 F and 1 R (see Reagents and Tools Table). These primers were 40 nucleotides in length, with one half corresponding to the beginning or end of the *URA3* gene and the other half to the flanking genomic sequences. The resulting PCR fragment enabled precise replacement of *ADE2* with *URA3*. A single isolate of such a construct was verified by sequencing and kept as a control Ura^+^ strain. DNA from it was used as a template in a PCR reaction that amplified the locus of interest together with flanking elements of 161 bp upstream of the START codon and 194 bp downstream of the STOP codon (2 F and 2 R primers were used for this purpose; see Reagents and Tools Table). The amplified fragment was used to transform all deletion strains of the BY4741 background using a lithium acetate-based method modified for 96-well titration plates (Gietz and Schiestl, 2007). For each deletion strain, a randomly selected single red-colored colony growing on uracil-deprived agar was picked and streaked to single cells. One of the resulting colonies was tested for the two expected trophic changes and was saved for future experiments. A list of 432 such modified strains is provided in Dataset EV1.

To obtain the final diploid test strains, pairs of BY4741 *MAT***a** Ura^+^ strains and BY4742 *MATα* Can^R^ strains carrying the same gene deletion were mated with each other. Diploids were detected on agar medium lacking uracil and adenine and then streaked to single cells on the same medium so that one of the resulting colonies could be randomly selected and stored. Two haploid wild-type strains yielded a diploid control strain. Dataset EV1 lists 392 pairs of the deletion strains that mated successfully. Both control and deletion diploids were heterozygous at the same two loci: *CAN1/can1* and *ADE2/ade2::URA3*. (DNA sequence analyses described below found that both the control strain and dozens of tested deletion strains were invariably *can1* when Can^R^ and *ade2::URA3* when Ura^+^, as expected).

### The LOH rate assay

The total of 392 diploid deletion strains was divided into five 96-well titration plates, each containing 12 wells filled with replicate cultures of the diploid control strain. These five “master” plates were kept frozen and only thawed briefly to inoculate the “working” plates. The latter were brought to the stationary phase and then used to inoculate the total of 20 replicate “mutant accumulation” cultures for each deletion strain, initially containing approximately 200 cells in 200 µL of fresh liquid medium. The thawing was done five times (separate time blocks), in which every master plate was used to inoculate four mutant accumulation cultures. In each time block, three screenings for LOH mutants were performed in parallel. In one of them, 10 µL aliquots of the mutant-accumulating stationary phase cultures were plated on SC agar containing both 100 mg/L canavanine and 1 g/L 5-FOA. In the other two assays, the same stationary phase cultures were diluted 1:100, and 5 µL of the dilutions were plated on agar containing either canavanine or 5-FOA. The fourth parallel assay was performed with non-selective SC agar to account for possible differences between the strains tested, not in LOH rate, but in cell density or plating efficiency. The mutant-accumulating plates were diluted so that 10 µL aliquots could be overlaid on plain SC agar, and the resulting colonies were relatively numerous but still discernible (up to 20–30 colonies per patch). All selective and non-selective plates were incubated together and photographed at 24, 48, 72, and 96 h to determine the time when the highest counts for individual strains could have been obtained. Several programs developed for colony counting were tested on our plates with 96 patches, but none gave reliable results. As a result, counting was done manually from enlarged photographs.

We used the microplate assay described above as the standard for the entire study because using separate agar plates for each deposition would require a prohibitive amount of plastic consumables and chemicals. There were ~1200 fluctuation assays, requiring a total of 27,180 replicate cultures. The control strain, BY4743, was replicated particularly frequently. However, we verified that this miniaturization was unlikely to affect mutant counts (see Fig. EV4).

Counts on the canavanine or 5-FOA plates were used to estimate the mutation rate to sLOH at either the *CAN1/can1* or *ADE2/ade2::URA3* loci for each deletion strain. We used the Drake method of calculating the mutation rate per cell division (Drake, 1991; Foster, 2006). As an approximation of the central tendency of the frequency, we used the median of the 20 replicate cultures. In the rare cases where the median was zero, we calculated the mean of the log-transformed non-zero counts, then reversed the transformation and averaged the result over 20 cultures. (To reduce the effect of jackpots.) Bootstrapping was then used to calculate confidence limits for mutation rates. Throughout the Results section of this manuscript, we only use the sLOH rate estimates obtained with the Drake method. However, we also explored the use of two other models: Salvador and *bz*-rates (Zheng, 2021; Gillet-Markowska et al, 2015). The estimates obtained using these models are presented and compared with those obtained using the Drake method in Dataset EV2. Drake and Salvador estimates are very close to each other; bz-rates estimates differ somewhat in absolute terms, but not in relative terms. They generally identify a similar order of strain instability. We then performed some of our key analyses, such as those in Fig. 2, using the three sets of estimates and found that they provided essentially the same conclusions. We are therefore confident in sticking to the relatively “crude” Drake method, which we considered to be the most appropriate from the outset, as it can be used when the number of mutants is high enough to reliably estimate their frequency. The other two methods excel when the number of mutants is low.

### Simulation of mutation–accumulation

We developed a Python program to simulate mutation–accumulation. It is available at GitHub as specified below. Initial code was generated using ChatGPT (OpenAI, GPT-4). The generated code was manually tested, modified, and validated to ensure accuracy and performance. Virtual populations were simulated from initial to final sizes equal to those propagated experimentally for specific deletion strains. A single cell produced two offspring, one of which could be randomly mutated at either of the still heterozygous loci, *CAN1/can1* or *ADE2/ade2::URA3* or both, with probabilities estimated as a corresponding sLOH mutation rate or their product, respectively. For each strain, 20 independent cultures (as in the experiment) were simulated to accumulate sLOH or dLOH mutations, and then the median and mean number of these mutants were calculated. The simulations were repeated four times, and the values of both parameters were averaged (we recognize that mutations leading to aneuploidy need not produce one normal and one mutant cell, but often only mutants, of which only one may be viable. However, leaving one progeny cell unchanged had little effect on the frequency of mutants because the first mutagenic divisions tended to occur when populations were in the thousands.

### Cytometric analysis of the DNA content

For this analysis, 43 deletion strains were selected from those with the highest rate of dLOH. Highly replicated mutant accumulation cultures were initiated for each of them, resulting in ~80–90 independent dLOH mutants (only one colony per replicate culture was harvested) on agar containing both canavanine and 5-FOA. Colonies (as complete as possible) were transferred to individual wells with fresh medium, allowed to grow overnight, and stored. For the control strain, 192 dLOH mutants were obtained in the same manner.

Just prior to the DNA content assays, 100 μL samples from master plates with dLOH mutants were taken, supplemented with 100 μL of fresh SC medium, and allowed to reach the stationary phase (the goal was to refresh and standardize the cultures while minimizing the number of cell divisions). Next, “cytometry” plates were assembled. A typical cytometry plate contained 84 dLOH mutants derived from a deletion strain, 8 replicate cultures of their parent deletion strain (i.e., not exposed to LOH screening), and two replicates of both the wild-type haploid BY4741 and the diploid BY4743. This arrangement allowed direct comparison of the dLOH mutants with their parental strain and helped to standardize the DNA content for each plate separately using the included wild-type strains. For some strains, the number of mutants was slightly less than 84; the absent mutants were replaced with additional replicates of the parent strain, so that the number of controls was occasionally higher than eight. The cytometry plates were subjected to the propidium iodide (PI) staining procedure (Sigma-Aldrich), and the amount of fluorescence of the dye was measured using a flow cytometer (CytoFLEX S, Beckman Coulter) with a blue laser at 488 nm and a PC5.5 filter (490/635 nm). Raw reads were then processed by gating intact single cells while discarding aggregates and fragmented cells. Gating was then restricted to cells in the G1 phase of the cell cycle. The median was used as a measure of central tendency of each sample.

### DNA sequence analysis

Trios of strains were used in this analysis: a parental deletion strain and two dLOH mutants derived from it. The two mutants were selected so one had a low and the other a high DNA content as determined by the cytometry assays. These two strains were randomly drawn from the lowest and highest five measurements of DNA content, respectively. The samples from the strains used in the cytometry analyses were thawed and had to be propagated to obtain enough cells for DNA extraction. DNA was isolated using Genomic Mini AX Yeast (A&A Biotechnology), and next-generation sequencing was performed by Novogene for high coverage sequencing (PE 150, expected read depth ~80X).

The resulting reads were mapped using BWA-MEM (Burrows-Wheeler Aligner for Maximum Exact Matches) (v0.717-r1188) (Li and Durbin, 2009) to a yeast reference genome: *S. cerevisiae* R64-1-1 supplemented with the kanMX4 cassette sequence. Samtools (v1.15.1) was used to sort, index, and analyze BAM file coverage (Danecek et al, 2021). Duplicate reads were marked using MarkDuplicates (Picard Toolkit 2019 v2.27.1). To detect possible aneuploidies, a coverage analysis was performed (samtools depth program with the -*a* parameter to obtain coverage for each position of the reference genome). Ploidy of chromosomes was estimated by dividing each chromosome into sections of 20 kb and calculating the average coverage for each compartment. Deviation from euploidy was checked by conducting an ANOVA test, with a Tukey post hoc test.

Detection of single nucleotide polymorphism and small (up to a few nucleotides) insertions and deletions was performed using Mutect2 software from GATK (Genome Analysis Toolkit) (v4.2.6.1). Uncertain reads were filtered out using the GATK FilterMutectCalls tool. Variants that were assigned a value of “PASS” or “germline” in the FILTER field were left for further analysis. Two analyses of the copy number variation were done with the programs CNVpytor v1.2 and CNVkit v0.9.10.. The VEP tool (v104) was used to annotate short variants. Structural variants were identified using smoove (v0.2.6), which performs analysis using the LUMPY tool (Layer et al, 2014). Annotation of structural variants and CNVs was performed using bedtools intersect (v2.30.0) and GTF files from Ensembl.

### Test of dLOH mutants for two additional markers

This experimental analysis used four deletion strains: *sem1*, *chl1*, *mre11*, and *rad27*. At least 100 double mutants (dLOH) were collected for each of them in the same way as described above. Care was taken to ensure that each mutant was isolated from a separate accumulation culture. These dLOH mutants were then raised separately in microcultures, diluted 1:100, and 10 µl of the dilution was transferred onto agar plates: SC-lysine or SD supplemented with uracil, leucine, histidine, and lysine (lacking methionine) and SD with all supplements, including methionine. Photographs taken after 24 and 48 h allowed differentiation between prototrophy and auxotrophy for lysine or methionine.

### Experimental evolution of dLOH mutants

To test whether departures from diploidy are stable, we selected those deletion strains that produced both significant increases and decreases in DNA content as estimated by the flow cytometry assay. There were 21 such strains plus control. For each of them, three dLOH mutants - one with the lowest, the second with the intermediate, and the third with the highest DNA content - were taken, replicated three times, and arranged so that the nine dLOH mutant cultures derived from a deletion strain occupied the same row within a 96-well plate. Cultures were serially transferred by inoculating 2 μL of stationary phase cells into 198 μL of fresh medium using the 96-pin automated pipette three times a week (2, 2, 3 days per cycle). Propagation continued for 63 days until 200 generations were obtained. The cultures from generations 0 and 200 were grown statically and subjected to periodical optical density (OD) measurements with a Tecan Infinite® 200 (the cultures were stirred prior to each measurement). Measurements covering the exponential phase of growth were used to calculate maximum growth rates and, after normalization by the maximum growth rate of the ancestral deletion strain, represented the relative fitness of individual strains. Cytometric analysis of DNA content for the 0 and 200 generation cultures was performed in the same manner as described above, using wild-type BY4743 as a standard.

## Supplementary information

Peer Review File

Dataset EV1

Dataset EV2

Dataset EV3

Dataset EV4

Dataset EV5

Dataset EV6

Dataset EV7

Dataset EV8

Dataset EV9

Source data Fig. 1

Source data Fig. 2

Source data Fig. 3

Source data Fig. 4

Source data Fig. 5

Expanded View Figures

## Data Availability

Illumina DNA sequencing fastq files from whole-genome sequencing of the ancestral deletion strains and dLOH mutants derived from them have been deposited in the NCBI Sequence Read Archive (SRA) under BioProject accession number PRJNA1153801. Computer program for the mutation–accumulation and a readme file are deposited at GitHub: https://github.com/AdrPirog/Sim_LOH_mut_acc. The source data of this paper are collected in the following database record: biostudies:S-SCDT-10_1038-S44320-026-00217-6.
