# Supplementary material for: Burst of aneuploidy: a cost of adaptation driven by breakdown of cell cycle control
Source: Mol Syst Biol. 2026 May 21;22(8):1292–311. doi: 10.1038/s44320-026-00217-6 (PMC13434691; doi:10.1038/s44320-026-00217-6)
Supplement: Supplementary file 1 [file 44320_2026_217_MOESM16_ESM.pdf]

Expanded View Figures

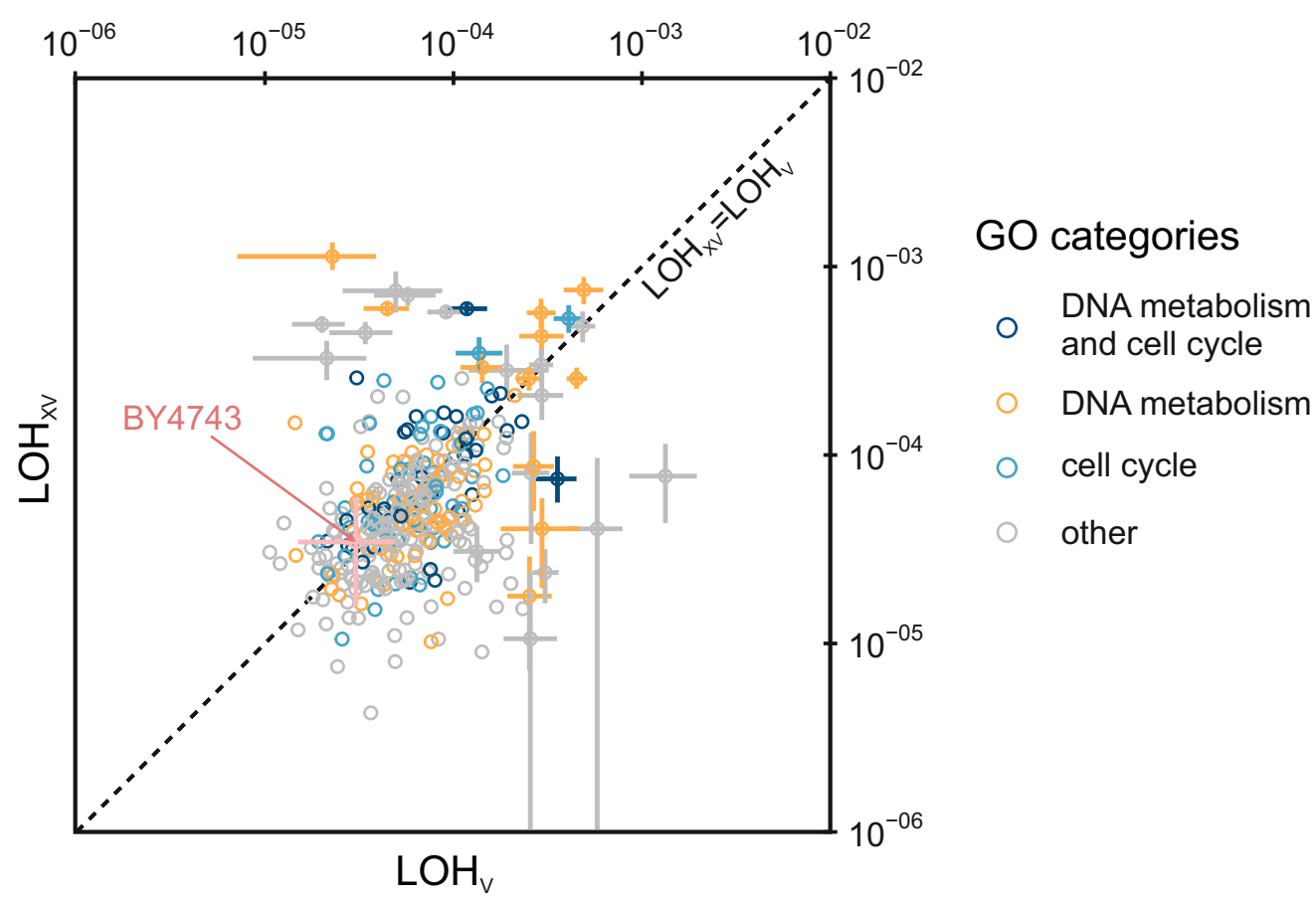

Figure EV1. Relation between sLOH rates.

Chromosome V (*CAN1/can1* locus) versus chromosome XV (*ADE2/ade2::URA3*) locus. Points represent the strains, $n = 393$, listed in Dataset EV2. Error bars refer to 95% confidence limits obtained through bootstrapping as described in Methods.

 © The Author(s)

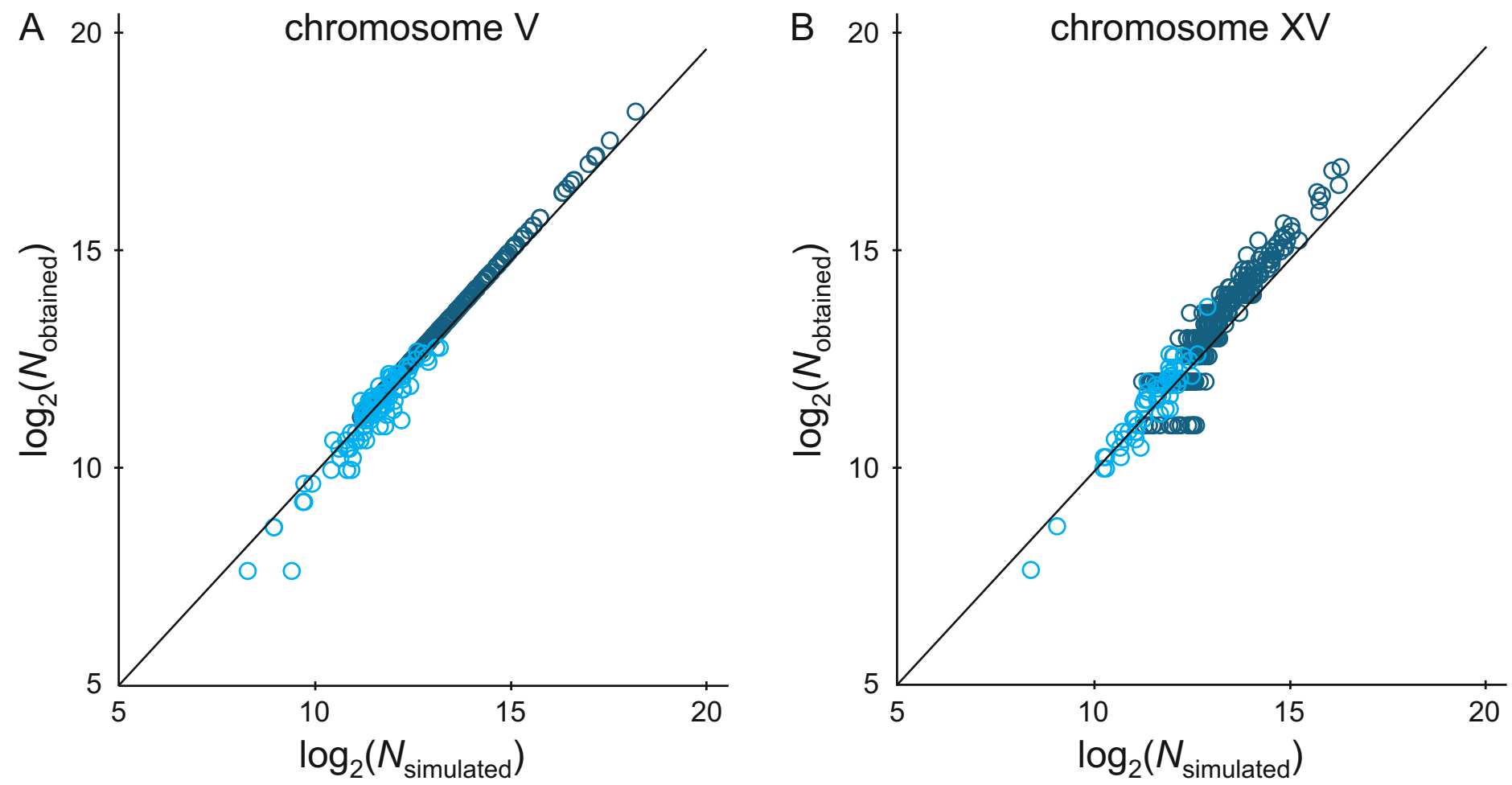

Figure EV2. The correlation between the obtained and expected numbers of sLOH mutants.

The correlation of sLOH mutants on chromosomes V (**A**) and XV (**B**) is shown across the set of studied deletion strains. The mutation rate μ of sLOH was calculated from the "obtained" (empirical) counts as described in the "Methods" section. These μ estimates were then used to simulate the mutational process in replicate cultures to obtain a simulated number of mutants. The numbers shown in the graphs are the median (dark) or mean (light) counts in the replicate cultures. The latter were used for cases with a low number of mutants, with the median being zero

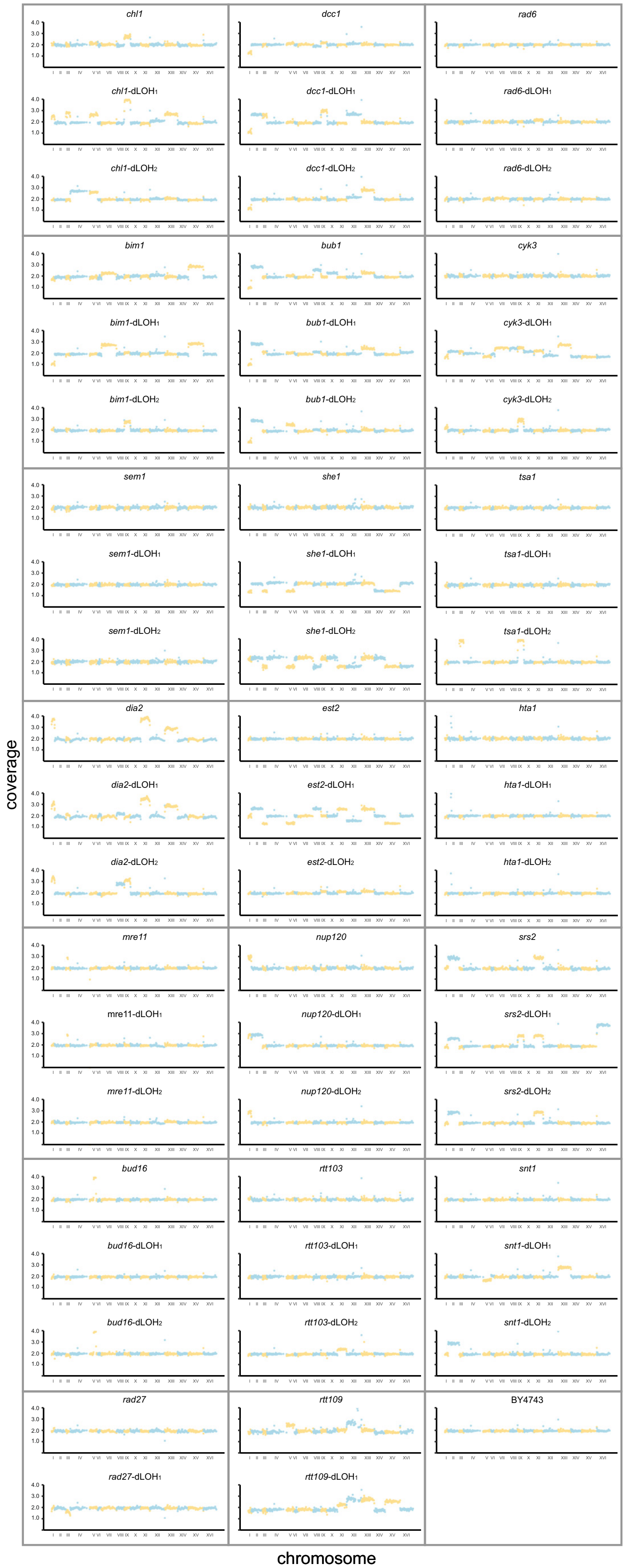
coverage
chromosome
chl1
chl1-dLOH1
chl1-dLOH2
dcc1
dcc1-dLOH1
dcc1-dLOH2
rad6
rad6-dLOH1
rad6-dLOH2
bim1
bim1-dLOH1
bim1-dLOH2
bub1
bub1-dLOH1
bub1-dLOH2
cyk3
cyk3-dLOH1
cyk3-dLOH2
sem1
sem1-dLOH1
sem1-dLOH2
she1
she1-dLOH1
she1-dLOH2
tsa1
tsa1-dLOH1
tsa1-dLOH2
dia2
dia2-dLOH1
dia2-dLOH2
est2
est2-dLOH1
est2-dLOH2
hta1
hta1-dLOH1
hta1-dLOH2
mre11
mre11-dLOH1
mre11-dLOH2
nup120
nup120-dLOH1
nup120-dLOH2
srs2
srs2-dLOH1
srs2-dLOH2
bud16
bud16-dLOH1
bud16-dLOH2
rtt103
rtt103-dLOH1
rtt103-dLOH2
snt1
snt1-dLOH1
snt1-dLOH2
rad27
rad27-dLOH1
rtt109
rtt109-dLOH1
BY4743

 © The Author(s)

◀ **Figure EV3. Coverage of DNA sequencing.**

Plots show the sum of DNA reads per 20 kb chromosome fragment, normalized to diploidy (or triploidy for *she1*).

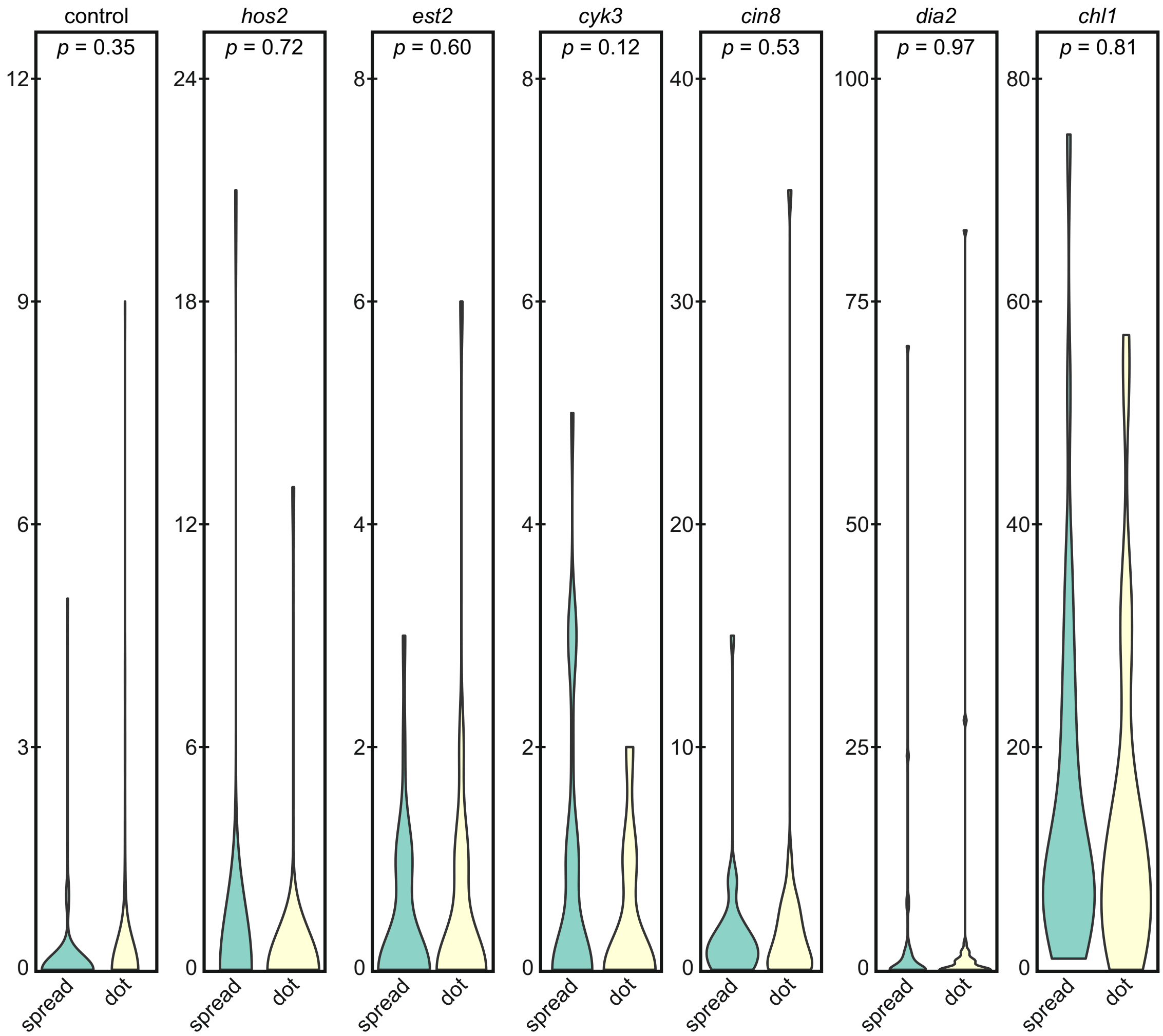

Figure EV4. The number of dLOH mutants in miniaturized and standard plate assays.

Samples were taken from the same replicate cultures in parallel and exposed to canavanine plus 5-FOA, either as whole-plate spreads or as dots that mimicked the conditions used in the miniaturized assay. The *P* values were obtained in a Wilcoxon's test comparing the scores obtained in the two assays for each gene separately.

 © The Author(s)
