# Supplementary material for: Burst of aneuploidy: a cost of adaptation driven by breakdown of cell cycle control
Source: Mol Syst Biol. 2026 May 21;22(8):1292–311. doi: 10.1038/s44320-026-00217-6 (PMC13434691; doi:10.1038/s44320-026-00217-6)
Supplement: Supplementary file 2 [file 44320_2026_217_MOESM1_ESM.pdf]

molecular systems biology

Burst of aneuploidy: A cost of adaptation driven by breakdown of cell cycle control

Adrian Pirog, Hanna Tutaj, Katarzyna Tomala, and Ryszard Korona

Corresponding author(s): Ryszard Korona (ryszard.korona@uj.edu.pl)

Review Timeline:

Submission Date:	18th Nov 25
Editorial Decision:	2nd Jan 26
Revision Received:	16th Feb 26
Editorial Decision:	3rd Apr 26
Revision Received:	17th Apr 26
Accepted:	30th Apr 26

Editor: Yehu Moran

Transaction Report:

(Note: With the exception of the correction of typographical or spelling errors that could be a source of ambiguity, letters and reports are not edited. Depending on transfer agreements, referee reports obtained elsewhere may or may not be included in this compilation. Referee reports are anonymous unless the Referee chooses to sign their reports.)

2nd Jan 2026

Manuscript Number: MSB-2025-13477
Title: Burst of aneuploidy: A cost of adaptation driven by breakdown of cell cycle control
Author: Adrian Pirog
Hanna Tutaj
Katarzyna Tomala
Ryszard Korona

Dear Dr Korona,

Thank you again for submitting your work to Molecular Systems Biology. We have now heard back from the three referees who agreed to evaluate your manuscript. As you will see from the reports below, the referees find the topic of your study of potential interest. They raise, however, substantial concerns on your work, which preclude its publication in its present form and require a thorough revision, should you be able to address all the major concerns in a satisfactory manner.

If you feel you can satisfactorily deal with the points listed by the referees, you may wish to submit a revised version of your manuscript. Please attach a covering letter giving details of the way in which you have handled each of the points raised by the referees. A revised manuscript will be once again subject to review and you probably understand that we can give you no guarantee at this stage that the eventual outcome will be favorable. Should you decide to revise your manuscript and wish to consult about any specific points please do not hesitate to contact me directly via email.

Yours sincerely,

Yehu Moran
Academic Editor
Molecular Systems Biology

We realize that it is difficult to revise to a specific deadline. In the interest of protecting the conceptual advance provided by the work, we recommend a revision within 3 months (2nd Apr 2026). Please discuss the revision progress ahead of this time with the editor if you require more time to complete the revisions. Use the link below to submit your revision:

https://msb.msubmit.net/cgi-bin/main.plex

IMPORTANT: Please read our guidelines for revised submissions: https://link.springer.com/journal/44320/submission-guidelines#cms-Revised-submissions

When you send your revision, we will require the following items:

1. the manuscript text in LaTeX, RTF or MS Word format
2. a letter with a detailed description of the changes made in response to the referees. Please specify clearly the exact places in the text (pages and paragraphs) where each change has been made in response to each specific comment given
3. three to four 'bullet points' highlighting the main findings of your study
4. a short 'blurb' text summarizing in two sentences the study (max. 250 characters)
5. a 'synopsis image' (550px width and max 400px height, Illustrator, PowerPoint or jpeg format), which can be used as 'visual title' for the synopsis section of your paper.
6. Please include an author contributions statement after the Acknowledgements section
7. Please complete the Author Checklist, which will be published alongside the paper as part of EMBO's transparent process.
8. When assembling figures, please refer to our figure preparation guideline in order to ensure proper formatting and readability in print as well as on screen:
https://media.springernature.com/original/springer-cms/rest/v1/content/27825798/data/v1
9. Please note that corresponding authors are required to supply an ORCID ID for their name upon submission of a revised manuscript.
10. At EMBO Press we ask authors to provide source data for the main manuscript figures. You will receive a separate email with instructions for providing source data with your revised manuscript, including how to upload and organize the files.
11. Include a Reagents and Tools Table as part of the Methods section, which can be downloaded from our author guidelines.

*** PLEASE NOTE *** As part of the EMBO Press transparent editorial process initiative (see our Editorial at https://dx.doi.org/10.1038/msb.2010.72), Molecular Systems Biology publishes online a Review Process File with each accepted manuscripts. This file will be published in conjunction with your paper and will include the anonymous referee reports, your point-by-point response and all pertinent correspondence relating to the manuscript. If you do NOT want this File to be published, please inform the editorial office at contact@molsystbiol.org within 14 days upon receipt of the present letter.

Reviewer #1:

This manuscript is an interesting and timely study that reports important findings on the cellular mechanisms controlling the emergence of complex genome rearrangements, using a two locus LOH selection system in diploid yeast deletion strains. The results are compelling and provide several novel observations, most notably the large excess of double LOH (dLOH) relative to expectations from independent single LOH (sLOH) events, the association with checkpoint and cell cycle related defects, and evidence that whole chromosome aneuploidy dominates the resulting genomic changes. Overall, the work has clear potential to advance the field, but several major concerns should be addressed to strengthen the manuscript. The English language should also be revised, as clarity and phrasing are not always optimal throughout the text.

Introduction
The statement "This means that selection favoring mutations in genes will often promote mutations in chromosomes instead" is not fully clear as written. If adaptation requires a specific amino acid change, a point mutation may appear more appropriate than LOH or aneuploidy, so the intended meaning should be clarified, for example by specifying the contexts in which chromosome level changes are more likely to provide a selectable effect.

The paragraph beginning "The selective pressures involved could be single or multiple" is somewhat vague and would benefit from clarification and a more concrete framing in the context of the experimental system used here.

Results
Mutation rate calculation
The Drake method provides a straightforward way to estimate mutation rates from frequency data. However, its accuracy relative to other approaches, such as the MSS maximum likelihood estimator, depends strongly on the number of mutation events observed in the experimental cultures. It would therefore be important to compare mutation rate estimates obtained with different methods to assess robustness and reproducibility across the dataset. It would also be important to correct for partial plating when estimating mutation rates.

sLOH rates
First, the authors should add a figure presenting their experimental system.

The sLOH results would benefit from a more thorough exploration. In particular, plotting both CAN1 and URA3 sLOH rates with their corresponding 95% confidence intervals for each deletion strain would greatly improve clarity. Such plots could also help address why some strains display extremely wide confidence intervals (for example mms22, shm2), while others show markedly asymmetric intervals (for example cyk3). An explicit discussion of the experimental or statistical origins of these patterns would be valuable.

In addition, a direct comparison of CAN1 versus URA3 sLOH rates, for example a scatter plot with an identity line, would allow assessment of whether sLOH rates are globally correlated across loci and would help identify deletion strains that behave as strong outliers with locus specific effects.

When stating that approximately 80% of deletion strains are more unstable than the control, it would be important to clarify whether this refers to both CAN1 and URA3 systems simultaneously, or to at least one of the two. A quick re analysis suggests that only about 70% of deletion strains show increased instability relative to the control in both systems.

dLOH frequencies
Dataset EV3 reports median dLOH frequencies for only a subset of deletion strains, with many strains lacking median values. The authors should clarify whether these missing medians correspond to zero values. If so, average frequencies are likely to be poor estimators of the true dLOH rate in these cases, as they are highly sensitive to jackpot events. Plating larger numbers of cells could help recover non zero median values for most strains. Deletion strains with null median values should either be excluded from Figure 1 or clearly flagged as such. In addition, a supplementary figure showing correlations between dLOH frequencies and individual as well as averaged sLOH rates would be a useful complement to the main analysis.

There is also a concern about the possibility of pre-existing mutants at the beginning of cultures. The authors inoculate 20 replicates with about 200 cells in 200 µL of SC medium without agitation. Given the high sLOH rates measured for some mutants

(Dataset EV2), it seems plausible that some cultures already contain pre-existing mutants. Since the authors report frequencies for double mutants rather than rates, this could influence the results and their interpretation. This concern is reinforced by the fact that in Figure 1 several mutants with high dLOH also have high sLOH (for example bub1, srs2, mre11). For some strains it may be preferable to repeat the experiment with a lower initial number of cells to validate the conclusions.

More generally, the manuscript compares rates for sLOH and frequencies for dLOH. It is not clear that this is methodologically optimal, since frequencies for dLOH are expected to be more affected by jackpot events than rates for sLOH, which could contribute to the reported excess of observed versus expected dLOH. In previous related studies (including Sampaio et al., Heasley et al., and Agier et al.), rates were estimated for double events using established fluctuation analysis frameworks. It would therefore be helpful to re-express the double LOH results in terms of rates as well, and then compare rates to rates, or frequencies to frequencies, to evaluate the impact of the current approach.

Excess of coincidental dLOH events
To estimate the expected number of coincidental dLOH events, the authors rely on computational simulations. First, the simulator code and the detailed expected values should be provided to enable full evaluation and reproducibility.

Second, the simulations assume that all dLOH mutants have zero fitness effects, arguing that the fitness spectrum of such mutants is unknown. This is a strong assumption that could be tested experimentally, for example by measuring growth rates for a representative set of sLOH and dLOH mutants across the 40 most unstable deletion strains.

The manuscript refers to previous work by Sampaio et al. (2020) and states that their observations of excess dLOH events were "dismissed as unsurprising." This wording seems overly strong and should be softened to more accurately reflect the tone and interpretation in the cited study. In addition, prior work by Heasley (Genetics 2020) and Sampaio (PNAS 2020) has shown that selection for sLOH can also be accompanied by additional genomic events, even if such events appear more frequent under double LOH selection. In this context, it is important that the authors control for the assumption that the two sLOH events occur independently. If the occurrence of one sLOH increases the probability of the second event, then the current calculation of the expected dLOH frequency, which assumes independence between events, would not be valid.

In Figure 2, it would be preferable to indicate the BY4743 reference clearly at the top of the figure rather than at the bottom. The authors should recall more directly earlier results from Agier et al. (2025), who reported an approximately 17 fold excess of double mutational events in wild type cells when accounting for both simultaneous and sequential accumulation, which seems closely related to the excess dLOH reported here for their BY4743 control strain.

Related to interpretation, the simulations suggest sequential events are much more frequent than simultaneous events (Figure 2, blue versus orange). However, the manuscript appears to exclude sequential scenarios too quickly, which may be premature. One plausible model is that an early mutation creates a transient mutator or hyper instability subpopulation, within which the second LOH then occurs. In this section it would be better to keep both hypotheses open, sequential and coincidental, and articulate what data would discriminate between them.

Finally, Figure 2 requires clarification. In the legend, the authors state that the white numbers specify how many times this exceeds the predicted frequency of simultaneous dLOH origin. However, for the first strain shown, the empirically measured median dLOH frequency is about 8.75×10^{-5}, while the simulated frequency appears around 1.0×10^{-5}, which does not seem consistent with the reported 176-fold increase. Similar discrepancies are observed for other deletion strains, including the 26-fold increase reported for the control strain. The authors should clarify how these ratios were calculated. Additionally, the meaning of the white label "e.o." should be defined explicitly in the figure legend.

GO categories
The authors indicate that three categories "DNA maintenance", "chromosome segregation" and "checkpoints" seem to be enriched among the most unstable strains. This could be quantified by running standard GO-term enrichment tests. It could also be interesting to perform similar enrichment analyses for the deletions that show the highest and lowest excess of dLOH compared to predicted frequencies. This aspect of the paper, namely the variable excess or apparent default (for example nup120, rad5, rad18) of observed dLOH relative to predicted frequencies, could be developed further because it may provide important hints on the cellular determinants controlling the observed bursts of genome instability.

Whole chromosome aneuploidy
The authors indicate that heterozygous positions were used to infer chromosome copy number. Dataset EV5 provides the list of heterozygous variants, but the corresponding analysis is not very clear and it is difficult to understand from the text how allele frequencies were used. The authors should explain more clearly what information was extracted from these signals and how it was used to conclude on chromosome copy numbers.

Burst of out of target aneuploidy
The authors convincingly show that the frequency of additional LOH is very high in four deletion strains by testing the LYS2 and MET15 markers on chromosomes II and XII. However, it would be important to perform the same phenotypic tests on a

comparable number of isolates derived directly from the parental deletion strains, to ensure that the increased LOH frequencies do not primarily result from the deleted gene in the parental strains rather than from association with dLOH events.

In addition, simultaneity is plausible because many non-selected aneuploidies are observed in double mutants. However, previous studies have also shown that selecting single LOH can produce additional events, even if they are more frequent under double selection. It would therefore be useful to test whether sLOH mutants also show additional, non-selected aneuploidies in this system. The same LYS2 and MET15 phenotypic tests could be performed on sLOH mutants to further confirm the specific-association of additional out of target aneuploidies with dLOH events.

Point mutations and segmental losses, gains or rearrangements
With short read sequencing, it may be difficult to conclude definitively on the absence of structural variants, so the wording should be more cautious. When stating that "smaller scale sequence alterations remain exceptionally rare," it would be clearer and more precise to say that point mutations, indels, or structural variants were not detected with the approach used here.

Experimental evolution
Despite the clear overall tendency toward diploidy after 200 generations for most strains, it would be very informative to sequence the genomes of a few strains to estimate relative chromosome coverage before and after the evolution step, in order to directly support the decline in aneuploidy suggested by the cellular DNA content measurements.

Discussion
The term 'genome rearrangements' may not be ideal in parts of the Discussion, since much of what is observed appears to be aneuploidization, whereas rearrangements can suggest recombination between DNA segments.

The sentence "Generalization appears inevitable" reads as overly teleological and would benefit from softer wording that avoids a Lamarckian tone.

The statement "It does not seem that these mutators experienced more frequent, multiple, independent genome alterations" is not fully convincing as written. By definition these are mutators relative to the reference, so they do experience more genome alterations. If the intended meaning is that they do not show more frequent independent, local events of the type that would explain the targeted double selection, this should be stated explicitly.

The notion of "non chromosomal mutations" is potentially ambiguous here. In this manuscript chromosomal often refers to aneuploidy, whereas in the cited work (Agier et al.) the relevant events include structural variants and point mutations that still occur on chromosomes. This terminology should be clarified to avoid confusion.

The statement "Bursts of aneuploidy may be considered an undesirable extreme, but they are the only way of generating broad genomic variation during asexual propagation" is somewhat reductive, because structural variants can also affect large fractions of the genome. This claim should be softened or reframed.

Finally, the last paragraph of the Discussion remains somewhat general and would benefit from a clearer articulation of testable predictions and limitations, particularly regarding the extent to which the yeast deletion mutants model transient failures of cell cycle control in other contexts.

Reviewer #2:

In this study, the authors test whether complex genome rearrangements arise gradually through independent events or instead occur in coordinated bursts, using double loss of heterozygosity (dLOH) at two loci on different chromosomes in diploid Saccharomyces cerevisiae as a model. By screening nearly 400 gene-deletion strains previously implicated in genome instability, they show that dLOH occurs far more frequently than predicted from independent single LOH events. Elevated dLOH frequencies are associated with deletions affecting cell-cycle checkpoints, DNA damage responses, and chromosome segregation. Molecular analyses sugest that dLOH mutants are dominated by whole-chromosome aneuploidy involving many non-selected chromosomes, with little contribution from point mutations, segmental copy-number variation, or recombination. The authors further argue that these deleterious aneuploid bursts are transient, regressing toward diploidy during experimental evolution, and conclude that selection for multiple adaptations tends to reveal systemic failures in mitotic control rather than locally targeted genome changes.

The authors make a compelling case that double LOH events occur more frequently than expected in a subset of yeast gene-disruption strains. However, several issues in the description of the experimental design, analysis, and interpretation weaken the strength of the overall conclusions.

First, the experimental setup requires clearer description to allow proper interpretation of the results. It is not clear how mutation accumulation was performed. Specifically, the phrase "20 replicate mutation accumulation cultures" is ambiguous: does this

mean that 20 independent cultures per strain were grown to stationary phase once, or that each strain underwent 20 sequential rounds of growth, dilution, and bottlenecking? If the latter, how many isolates were carried forward for each mutant background? A clearer explanation is needed to understand how many generations elapsed before the dLOH assay and whether this was consistent across strains.

Second, while the simulation-based analysis is conceptually appealing, it is difficult to evaluate without a clearer description of the model or access to the code used to implement it. The manuscript does not indicate whether the code is publicly available, how it was validated, or whether comparable simulation frameworks exist and were used for benchmarking. These details are particularly important because key conclusions depend on the magnitude of the excess of observed dLOH over expected values.

Third, although the set of mutant strains examined is interesting, it complicates the interpretation of any enrichment analyses. The tested gene set is already heavily enriched for genome instability and cell-cycle-related functions. As a result, conclusions regarding the importance of cell-cycle control would require enrichment tests performed against this specific background rather than the genome as a whole. Alternatively, a randomly selected deletion set would need to be subjected to the same mutant accumulation and dLOH assays to establish an appropriate baseline.

Beyond these issues, there are more substantial concerns with the remaining analyses. The authors use flow cytometry (FACS) to assess DNA content before and after selection for dLOH, then select isolates from high- and low-DNA-content bins for genome sequencing. In some cases (e.g., cyk3), this approach yields aneuploid strains with chromosome gains or losses consistent with FACS measurements. In other cases (e.g., mre11), isolates recovered from both high- and low-content bins appear largely euploid. There is no clear relationship between FACS variance and the resulting ploidy states, and it is unclear what hypothesis this selection scheme is intended to test. As presented, the analysis appears to select for ploidy anomalies and then argue that these anomalies explain dLOH, rather than testing whether dLOH generally coincides with genome-wide instability. A more appropriate approach would be to analyze randomly selected dLOH isolates from each genetic background for genomic abnormalities, ideally using orthogonal karyotype assays such as chromosome-specific qPCR. Importantly, the same genomic analyses should also be performed on multiple dLOH isolates derived from the wild-type diploid background.

Minor issues

Figure 2: The fold-excess values of observed versus expected dLOH are likely too small to be legible in a published figure. These values should be presented in an accompanying table.

Figure 3: The horizontal reference line appears to represent a median value, but this is not explicitly indicated in the figure or legend.

Figure 4: The term "spontaneously arising polymorphic variants" is insufficiently explained. Despite careful reading of the figure legend, Methods, and Results, it remains unclear how these variants are defined, detected, or used in the analysis. Additional explanation is required.

Reviewer #1:

This manuscript is an interesting and timely study that reports important findings on the cellular mechanisms controlling the emergence of complex genome rearrangements, using a two locus LOH selection system in diploid yeast deletion strains. The results are compelling and provide several novel observations, most notably the large excess of double LOH (dLOH) relative to expectations from independent single LOH (sLOH) events, the association with checkpoint and cell cycle related defects, and evidence that whole chromosome aneuploidy dominates the resulting genomic changes. Overall, the work has clear potential to advance the field, but several major concerns should be addressed to strengthen the manuscript. The English language should also be revised, as clarity and phrasing are not always optimal throughout the text.

<<**AUTHORS**: We are grateful for the generally positive evaluation of our work and the detailed comments on our manuscript. We have made every effort to address these comments, and we believe that we have succeeded. In particular, we add several new figures, datasheets and statistical analyses, as listed below.>>

Introduction

The statement "This means that selection favoring mutations in genes will often promote mutations in chromosomes instead" is not fully clear as written. If adaptation requires a specific amino acid change, a point mutation may appear more appropriate than LOH or aneuploidy, so the intended meaning should be clarified, for example by specifying the contexts in which chromosome level changes are more likely to provide a selectable effect.
<<**AUTHORS**: The statement in question is rewritten; **lines 45-47**.>>

The paragraph beginning "The selective pressures involved could be single or multiple" is somewhat vague and would benefit from clarification and a more concrete framing in the context of the experimental system used here.
<<**AUTHORS**: This sentence is deleted as it was misleading and somewhat redundant after the preceding sentence. The preceding and following sentences are slightly modified; **lines 51-54**.>>

Results
Mutation rate calculation
The Drake method provides a straightforward way to estimate mutation rates from frequency data. However, its accuracy relative to other approaches, such as the MSS maximum likelihood estimator, depends strongly on the number of mutation events observed in the experimental cultures. It would therefore be important to compare mutation rate estimates obtained with different methods to assess robustness and reproducibility across the dataset. It would also be important to correct for partial plating when estimating mutation rates.
<<**AUTHORS**: We paid a lot of attention to this question. Although we did not mention it in the initial msc, we completed the rate calculations with two frequently used programs: rSalvador (full MLE model) and *bz*-rates (Ma-Sandri-Sarkar method). In the present msc, we provide a supplementary table containing the results obtained with these two methods along those derived using Drake's method; **Dataset EV2**. It must be stressed that we had to use 200 µl cultures to

obtain any dLOH mutants, which are rare, but this also meant that sLOH mutants occurred in the thousands in a single mutation-accumulation culture. Both the rSalvador and *bz*-rates models are designed to study setups in which mutational events are few; both are only approximations tailored to work specifically in such situations. Let us consider the correction for partial plating provided by bz-rates. We only had to plate 1/4000 of the cultures on single selective media, and we are convinced that the correction malfunctioned. Cells and liquids are physical entities, not abstract numerical values. Conversely, plating around 10,000 cells and identifying a few (or dozens) of them is straightforward, making Drake the most obvious choice; **lines 502-513**.>>

First, the authors should add a figure presenting their experimental system.
<<**AUTHORS**: We have now added such a graph as **Fig. 6**. It is placed in the 'Materials and Methods' section rather than at the beginning of 'Results' because it is fairly complex and some readers may not wish to engage with it before learning the most important findings, **lines 420-421**.>>

The sLOH results would benefit from a more thorough exploration. In particular, plotting both CAN1 and URA3 sLOH rates with their corresponding 95% confidence intervals for each deletion strain would greatly improve clarity. Such plots could also help address why some strains display extremely wide confidence intervals (for example mms22, shm2), while others show markedly asymmetric intervals (for example cyk3). An explicit discussion of the experimental or statistical origins of these patterns would be valuable. In addition, a direct comparison of CAN1 versus URA3 sLOH rates, for example a scatter plot with an identity line, would allow assessment of whether sLOH rates are globally correlated across loci and would help identify deletion strains that behave as strong outliers with locus specific effects.
<<**AUTHORS**: We now provide such a plot as supplementary **Figure EV1**. For most deletion strains, the sLOH rates obtained for chromosomes V and XV form an extended hump along a diagonal. There are outliers, but this does not necessarily indicate that some strains are unstable with respect to a single chromosome. Deletion strains are often 'sick'; their metabolism is distorted in a way that can render counterselection against *CAN1* or *URA3* ineffective or overly effective. For example, *shm2* is defective in the generation of pyrimidine precursors, which is likely why it has extremely high sLOH scores at *URA3*. Regarding the width of the confidence limits of the mutation rate estimates, we optimized our protocol to narrow them as much as possible for the most unstable strains, which are of the most interest to us, and we generally succeeded. When mutation rates are low and mutant scores are highly variable, estimates tend to be imprecise. Please note that we had as many as 1,024 replicates for the control strain, which allowed us to properly anchor all our calculations. However, we decided that such effort and material expense was not justified for strains with inconspicuous instability; **lines 132-138**.>>

When stating that approximately 80% of deletion strains are more unstable than the control, it would be important to clarify whether this refers to both CAN1 and URA3 systems simultaneously, or to at least one of the two. A quick reanalysis suggests that only about 70% of deletion strains show increased instability relative to the control in both systems.
<<**AUTHORS**: We now provide an exact proportion of strains with sLOH rates higher than the control, 68% for *CAN1*, 82% for *URA3*, 42% for both, which is still impressive as we argue in the msc; **lines 123-127**.>>

dLOH frequencies
Dataset EV3 reports median dLOH frequencies for only a subset of deletion strains, with many strains lacking median values. The authors should clarify whether these missing medians correspond to zero values. If so, average frequencies are likely to be poor estimators of the true dLOH rate in these cases, as they are highly sensitive to jackpot events. Plating larger numbers of cells could help recover non zero median values for most strains. Deletion strains with null median values should either be excluded from Figure 1 or clearly flagged as such.
<<**AUTHORS**: Figure 1 is based solely on average frequencies to make it consistent for all strains. It is now clearly stated in the text and figure description. Both averages and medians are listed in **Dataset EV3**.

In addition, a supplementary figure showing correlations between dLOH frequencies and individual as well as averaged sLOH rates would be a useful complement to the main analysis.
<<**AUTHORS**: We could easily provide such a graph, however our work is already heavily loaded with supplementary material. We propose that a report of a statistical test (Spearman's r=0.61; n=314; p=2.2e-16) is sufficient here; **lines 136-138**.>>

There is also a concern about the possibility of pre-existing mutants at the beginning of culture. The authors inoculate 20 replicates with about 200 cells in 200 µL of SC medium without agitation. Given the high sLOH rates measured for some mutants (Dataset EV2), it seems plausible that some cultures already contain pre-existing mutants. Since the authors report frequencies for double mutants rather than rates, this could influence the results and their interpretation. This concern is reinforced by the fact that in Figure 1 several mutants with high dLOH also have high sLOH (for example bub1, srs2, mre11). For some strains it may be preferable to repeat the experiment with a lower initial number of cells to validate the conclusions.
<<**AUTHORS**: Given the instability of the strains, the question of mutants within the inoculum appears reasonable. The inoculum size is now presented also in **Fig. 6**. We are confident that it was small enough to not distort our results. The rate of sLOH in the most unstable strains remained well below 1/200, meaning that even a single cell in the inoculum is unlikely. Furthermore, our mutant counts, particularly the means and medians, are well below 2E06, which is 1/200 of the approximate number of cells per mutation accumulation culture, if there was at least one mutant cell to begin with, >>

More generally, the manuscript compares rates for sLOH and frequencies for dLOH. It is not clear that this is methodologically optimal, since frequencies for dLOH are expected to be more affected by jackpot events than rates for sLOH, which could contribute to the reported excess of observed versus expected dLOH. In previous related studies (including Sampaio et al., Heasley et al., and Agier et al.), rates were estimated for double events using established fluctuation analysis frameworks. It would therefore be helpful to re-express the double LOH results in terms of rates as well, and then compare rates to rates, or frequencies to frequencies, to evaluate the impact of the current approach.
<<**AUTHORS**: In our initial analyses, we calculated rates of dLOH. However, we then realized that a dLOH mutant could have arisen in one of two ways: either all at once, which would be the true dLOH rate; or as a sequence of two sLOH events within a growing pool of sLOH mutants. Including all dLOH events would improperly inflate the dLOH rate. This is why we only

calculate sLOH rates and then use these to determine the origin of dLOH mutants in computer simulations. We aim to articulate our differences with Samapio et al. more clearly, while maintaining a respectful and constructive tone; **lines 179-184**. >>

Excess of coincidental dLOH events

To estimate the expected number of coincidental dLOH events, the authors rely on computational simulations. First, the simulator code and the detailed expected values should be provided to enable full evaluation and reproducibility.

<<**AUTHORS**: The code is deposited at GitHub, with address provided, **line 618**. Furthermore, we provide supplementary **Table EV4** which contains the 'expected values', that is, results of our simulations which hopefully makes the simulations more understandable.>

Second, the simulations assume that all dLOH mutants have zero fitness effects, arguing that the fitness spectrum of such mutants is unknown. This is a strong assumption that could be tested experimentally, for example by measuring growth rates for a representative set of sLOH and dLOH mutants across the 40 most unstable deletion strains

<<**AUTHORS**: In the previous msc, we admitted that the fitness effects of the thousands of mutants could not be estimated, but setting them to zero is actually the most conservative approach in our case, **lines 151-162**. In the present msc, we report simulations in which we set the costs of sLOH and dLOH mutants to arbitrary values. The results are clear and reinforce our point (see **Dataset EV5**).

The manuscript refers to previous work by Sampaio et al. (2020) and states that their observations of excess dLOH events were "dismissed as unsurprising." This wording seems overly strong and should be softened to more accurately reflect the tone and interpretation in the cited study. In addition, prior work by Heasley (Genetics 2020) and Sampaio (PNAS 2020) has shown that selection for sLOH can also be accompanied by additional genomic events, even if such events appear more frequent under double LOH selection. In this context, it is important that the authors control for the assumption that the two sLOH events occur independently. If the occurrence of one sLOH increases the probability of the second event, then the current calculation of the expected dLOH frequency, which assumes independence between events, would not be valid

<<**AUTHORS**: We consider that our original wording reflected appropriate caution, as indicated by the use of qualifying terms such as "could be." Nevertheless, in the revised msc, we have removed the questioned phrasing and included the statement, "our results are consistent with earlier reports"; **lines 187-191**.>>

In Figure 2, it would be preferable to indicate the BY4743 reference clearly at the top of the figure rather than at the bottom.

<<**AUTHORS**: It would be likely helpful to have BY4743 at the top but we have to split the figure into sections with different scales of frequency otherwise the most unstable strains would dwarf all others. BY4743 is much less unstable than the top ones and therefore it must be at the bottom, although we now redraw the graph to make it more readable. There is specific comment on that in **Fig. 2** legend written in italics, *Scale bar*, **line 760**.

The authors should recall more directly earlier results from Agier et al. (2025), who reported an approximately 17 fold excess of double mutational events in wild type cells when accounting for both simultaneous and sequential accumulation, which seems closely related to the excess dLOH reported here for their BY4743 control strain.
<<**AUTHORS**: We now refer to Agier et al. more thoroughly, **lines 379-384**.>>

Related to interpretation, the simulations suggest sequential events are much more frequent than simultaneous events (Figure 2, blue versus orange). However, the manuscript appears to exclude sequential scenarios too quickly, which may be premature. One plausible model is that an early mutation creates a transient mutator or hyper instability subpopulation, within which the second LOH then occurs. In this section it would be better to keep both hypotheses open, sequential and coincidental, and articulate what data would discriminate between them.
<<**AUTHORS**: We do not dismiss sequential scenarios too quickly; the purpose of computer simulations is precisely to address that possibility. However, we have identified the fragment that probably prompted the reviewer's comment and rewritten it; **lines 275-285**.>>

Finally, Figure 2 requires clarification. In the legend, the authors state that the white numbers specify how many times this exceeds the predicted frequency of simultaneous dLOH origin. However, for the first strain shown, the empirically measured median dLOH frequency is about 8.75×10^{-5}, while the simulated frequency appears around 1.0×10^{-5}, which does not seem consistent with the reported 176-fold increase. Similar discrepancies are observed for other deletion strains, including the 26-fold increase reported for the control strain. The authors should clarify how these ratios were calculated. Additionally, the meaning of the white label "e.o." should be defined explicitly in the figure legend.
<<**AUTHORS**: We stand by our conclusions but will now explain them in a hopefully clearer way; **lines 168-176**.>>

GO categories

The authors indicate that three categories "DNA maintenance", "chromosome segregation" and "checkpoints" seem to be enriched among the most unstable strains. This could be quantified by running standard GO-term enrichment tests. It could also be interesting to perform similar enrichment analyses for the deletions that show the highest and lowest excess of dLOH compared to predicted frequencies. This aspect of the paper, namely the variable excess or apparent default (for example nup120, rad5, rad18) of observed dLOH relative to predicted frequencies, could be developed further because it may provide important hints on the cellular determinants controlling the observed bursts of genome instability.
<<**AUTHORS**: There are now three sheets in **Dataset EV6** entitled 'GO cycle checkpoints', 'GO term enrichment' and ‘ranked list’. The second sheet provides an extensive standard enrichment test and the msc contains now a reference to it. As is often the case with such analyses, lots of categories were identified and these can be inspected by the reader. We maintain our view that identifying cell cycle checkpoints is justified and informative.>>

Whole chromosome aneuploidy

The authors indicate that heterozygous positions were used to infer chromosome copy number. Dataset EV5 provides the list of heterozygous variants, but the corresponding analysis is not very clear and it is difficult to understand from the text how allele frequencies were used. The authors

should explain more clearly what information was extracted from these signals and how it was used to conclude on chromosome copy numbers.
<<**AUTHORS**: Indeed, clarification is needed. We can achieve this by analyzing an example strain, *dia2*; **lines 244-251**.>>

Burst of out of target aneuploidy
The authors convincingly show that the frequency of additional LOH is very high in four deletion strains by testing the *LYS2* and *MET15* markers on chromosomes II and XII. However, it would be important to perform the same phenotypic tests on a comparable number of isolates derived directly from the parental deletion strains, to ensure that the increased LOH frequencies do not primarily result from the deleted gene in the parental strains rather than from association with dLOH events. In addition, simultaneity is plausible because many non-selected aneuploidies are observed in double mutants. However, previous studies have also shown that selecting single LOH can produce additional events, even if they are more frequent under double selection. It would therefore be useful to test whether sLOH mutants also show additional, non-selected aneuploidies in this system. The same LYS2 and MET15 phenotypic tests could be performed on sLOH mutants to further confirm the specific-association of additional out of target aneuploidies with dLOH events.
<<**AUTHORS**: Our main experiment was designed to examine two chromosomal locations because only *CAN1* and *URA3* provide an effective distinction between LOH and non-LOH cells. The other two markers were then used in a limited scope in a follow-up experiment to confirm the results of the DNA sequence analysis, which suggested multiple aneuploidies based on statistical arguments alone, albeit strong ones. Simple classic genetic data applied to chromosomes II and XII confirmed this nicely. If confirmation was not that clear, further experiments as suggested by the reviewer would be desirable.>>

Point mutations and segmental losses, gains or rearrangements
With short read sequencing, it may be difficult to conclude definitively on the absence of structural variants, so the wording should be more cautious. When stating that "smaller scale sequence alterations remain exceptionally rare," it would be clearer and more precise to say that point mutations, indels, or structural variants were not detected with the approach used here.
<<**AUTHORS**: Ok, we are now more cautious; **lines 304-306**. >>

Experimental evolution
Despite the clear overall tendency toward diploidy after 200 generations for most strains, it would be very informative to sequence the genomes of a few strains to estimate relative chromosome coverage before and after the evolution step, in order to directly support the decline in aneuploidy suggested by the cellular DNA content measurements.
<<**AUTHORS**: We agree that more data could provide further insight. However, our goal was strictly to show the "clear overall tendency towards diploidy" identified by the reviewer. Please note that we started this experiment to support the conclusion from the sequence data that there were 'non-integer' chromosomes, e.g. 2.3, which we interpreted as evidence of a mixture of diploid and triploid chromosomes, with the latter probably receding. The general prediction was that there would be coalescence towards diploidy, and this was satisfactorily confirmed. >>

Discussion

The term 'genome rearrangements' may not be ideal in parts of the Discussion, since much of what is observed appears to be aneuploidization, whereas rearrangements can suggest recombination between DNA segments.
<<**AUTHORS**: Indeed, readers might tend to think about within-chromosome rearrangements. We now avoid this term replacing it with “changes” or “alterations” whenever appropriate.>>

The sentence "Generalization appears inevitable" reads as overly teleological and would benefit from softer wording that avoids a Lamarckian tone.
<<**AUTHORS**: Deleted.>>

The statement "It does not seem that these mutators experienced more frequent, multiple, independent genome alterations" is not fully convincing as written. By definition these are mutators relative to the reference, so they do experience more genome alterations. If the intended meaning is that they do not show more frequent independent, local events of the type that would explain the targeted double selection, this should be stated explicitly.
<<**AUTHORS**: To address this comment, the whole paragraph is rewritten and shortened; **lines 363-375**.>>

The notion of "non chromosomal mutations" is potentially ambiguous here. In this manuscript chromosomal often refers to aneuploidy, whereas in the cited work (Agier et al.) the relevant events include structural variants and point mutations that still occur on chromosomes. This terminology should be clarified to avoid confusion.
<<**AUTHORS**: That’s right, we now list the type mutations found in that work, **lines 379-384**.

The statement "Bursts of aneuploidy may be considered an undesirable extreme, but they are the only way of generating broad genomic variation during asexual propagation" is somewhat reductive, because structural variants can also affect large fractions of the genome. This claim should be softened or reframed.
AUTHORS: This sentence is now deleted, the paragraph starts with: “Paradoxically, our results ...” and it finishes our Discussion.

Finally, the last paragraph of the Discussion remains somewhat general and would benefit from a clearer articulation of testable predictions and limitations, particularly regarding the extent to which the yeast deletion mutants model transient failures of cell cycle control in other contexts.
<<**AUTHORS:** We have tried to be clearer in the revised manuscript. However, such remarks will always be necessarily hypothetical. We just hope that a few of the last sentences in the text can be a bit more general, perhaps inspiring someone to come up with new ideas, **lines 401-414.**

Reviewer #2:

In this study, the authors test whether complex genome rearrangements arise gradually through independent events or instead occur in coordinated bursts, using double loss of heterozygosity (dLOH) at two loci on different chromosomes in diploid Saccharomyces cerevisiae as a model. By screening nearly 400 gene-deletion strains previously implicated in genome instability, they show that dLOH occurs far more frequently than predicted from independent single LOH events.

Elevated dLOH frequencies are associated with deletions affecting cell-cycle checkpoints, DNA damage responses, and chromosome segregation. Molecular analyses suggest that dLOH mutants are dominated by whole-chromosome aneuploidy involving many non-selected chromosomes, with little contribution from point mutations, segmental copy-number variation, or recombination. The authors further argue that these deleterious aneuploid bursts are transient, regressing toward diploidy during experimental evolution, and conclude that selection for multiple adaptations tends to reveal systemic failures in mitotic control rather than locally targeted genome changes.

The authors make a compelling case that double LOH events occur more frequently than expected in a subset of yeast gene-disruption strains. However, several issues in the description of the experimental design, analysis, and interpretation weaken the strength of the overall conclusions.

<<**AUTHORS**: We are pleased to learn that the reviewer found our work convincing in its main message. The accompanying comments were incisive and helpful in improving the manuscript.>>

First, the experimental setup requires clearer description to allow proper interpretation of the results. It is not clear how mutation accumulation was performed. Specifically, the phrase "20 replicate mutation accumulation cultures" is ambiguous: does this mean that 20 independent cultures per strain were grown to stationary phase once, or that each strain underwent 20 sequential rounds of growth, dilution, and bottlenecking? If the latter, how many isolates were carried forward for each mutant background? A clearer explanation is needed to understand how many generations elapsed before the dLOH assay and whether this was consistent across strains.
<<**AUTHORS**: It is indeed difficult to follow all the experimental and computational analyses we completed. We have now introduced a diagram depicting all the stages of our research, **Fig. 6**>

Second, while the simulation-based analysis is conceptually appealing, it is difficult to evaluate without a clearer description of the model or access to the code used to implement it. The manuscript does not indicate whether the code is publicly available, how it was validated, or whether comparable simulation frameworks exist and were used for benchmarking. These details are particularly important because key conclusions depend on the magnitude of the excess of observed dLOH over expected values.
<<**AUTHORS**: The code has now been deposited on GitHub; **line 618**. Furthermore, we provide a supplementary table (**Dataset EV4**) showing the results of the simulations, which should help to clarify what we have done.>>

Third, although the set of mutant strains examined is interesting, it complicates the interpretation of any enrichment analyses. The tested gene set is already heavily enriched for genome instability and cell-cycle-related functions. As a result, conclusions regarding the importance of cell-cycle control would require enrichment tests performed against this specific background rather than the genome as a whole. Alternatively, a randomly selected deletion set would need to be subjected to the same mutant accumulation and dLOH assays to establish an appropriate baseline.

<<**AUTHORS**: Following this advice, we performed a test for enrichment within the ranked list of the set of four hundred studied genes. This showed that the terms ‘cell cycle checkpoint’ and ‘negative control of cell cycle’ are enriched among the most unstable, even within this specific sample of genes. The test has been added as a new spreadsheet ‘ranked list’, together with a 'readme' sheet, to the **Dataset EV6**, to guide readers through GO analyses.>>

Beyond these issues, there are more substantial concerns with the remaining analyses. The authors use flow cytometry (FACS) to assess DNA content before and after selection for dLOH, then select isolates from high- and low-DNA-content bins for genome sequencing. In some cases (e.g., cyk3), this approach yields aneuploid strains with chromosome gains or losses consistent with FACS measurements. In other cases (e.g., mre11), isolates recovered from both high- and low-content bins appear largely euploid. There is no clear relationship between FACS variance and the resulting ploidy states, and it is unclear what hypothesis this selection scheme is intended to test. As presented, the analysis appears to select for ploidy anomalies and then argue that these anomalies explain dLOH, rather than testing whether dLOH generally coincides with genome-wide instability.

<<**AUTHORS**: We have now introduced a new test and summary of its results: “We also tested whether the extent of DNA gains or losses (as measured by F) correlated with the frequency of dLOH. However, no such association was observed (Spearman's rs=0.162, n=44, p=0.255). This could mean that aneuploidization did not occur in all of the most unstable strains, and that others may have acquired different changes to their genomes, such as point mutations.”; **lines 219-223**. We say it after obtaining cytometric data when seeing that some strains remained close to diploidy could mean that changes other (smaller) than aneuploidization could dominate in producing double LOHs. It was after learning the sequence DNA when we could see that some chromosomes were close to diploidy (1.8, 2.3) but nevertheless departed from it in statistical terms implying the mixture of clones with an aneuploidy in recession, **Fig 4**.>>

A more appropriate approach would be to analyze randomly selected dLOH isolates from each genetic background for genomic abnormalities, ideally using orthogonal karyotype assays such as chromosome-specific qPCR.

<<**AUTHORS**: From a statistical point of view, we agree that sequencing a large number of randomly selected strains would be the most accurate approach. However, due to financial constraints, we opted to select a few strains that were either close to or far from diploidy. As mentioned in the previous answer, we were fortunate to demonstrate that even mutants close to diploidy showed clear signs of transient and receding aneuploidy. In this way, the rather restricted scope of our sample still led to a clear answer: aneuploidy was the dominant phenomenon behind dLOH.

A related question is the selection of strains for the evolution experiments in which we transferred batch cultures for 200 generations to see whether variation in the DNA content as determined by flow cytometry tends to collapse towards diploidy. We now explain that our selections were not arbitrary: “This was done for 21 original deletion strains, which generally had the highest frequency of dLOH mutants. Trios of mutants were selected for each deletion strain. The aim was to achieve a wide range of departures from diploidy, but three dLOH mutants with the highest and three with the lowest cytometry scores were excluded as they were potentially unrepresentative. We then identified three groups of five mutants with the lowest,

medium and highest DNA content, randomly selecting one mutant from each group; **lines 320-325**.>>

Importantly, the same genomic analyses should also be performed on multiple dLOH isolates derived from the wild-type diploid background.
<<**AUTHORS**: We understand that this comment relates to the experiment in which LOH events were phenotypically detected at four chromosomes, **lines 363-375**. We did not plan to analyze the sequence of those mutants. This was an additional experiment performed only after we observed that the sequenced mutants had affected many 'off-target' chromosomes, i.e. those not carrying selective markers. Based on the sequence data displayed in **Fig. 4**, we reasoned that, if off-target chromosomes are involved as often as it appears, we could select for dLOH on the V and XV chromosomes and observe that the II and XII chromosomes were often affected too. They were indeed affected. The findings summarized in **Fig. 4** are so statistically significant that this corroboration may appear unnecessary. Nevertheless, we suggest that this additional experiment remain in the manuscript, as it may convince some readers of our main claim that two-point selection often leads to multi-point restructuring.>>

Minor issues

Figure 2: The fold-excess values of observed versus expected dLOH are likely too small to be legible in a published figure. These values should be presented in an accompanying table.
<<**AUTHORS**: we now have a sheet on that in **Dataset EV5**, with a reference to it in **line 758**, that is, Fig. 2 description.

Figure 3: The horizontal reference line appears to represent a median value, but this is not explicitly indicated in the figure or legend.
<<**AUTHORS**: now added it in **Fig. 3** legend.>>

Figure 4: The term "spontaneously arising polymorphic variants" is insufficiently explained. Despite careful reading of the figure legend, Methods, and Results, it remains unclear how these variants are defined, detected, or used in the analysis. Additional explanation is required.
<<**AUTHORS**: The questioned sentence in the **Fig. 4** legend has been rewritten for clarity. To make the matter clear, we added in the main text an example of strain dia2 to explain how these variants were used alongside the DNA coverage to infer ploidy, **lines 244-251**.

3rd Apr 2026

Manuscript Number: MSB-2025-13477R
Title: Burst of aneuploidy: A cost of adaptation driven by breakdown of cell cycle control
Author: Adrian Pirog
Hanna Tutaj
Katarzyna Tomala
Ryszard Korona

Dear Dr Korona,

Thank you again for submitting your work to Molecular Systems Biology. We have now heard back from the three referees who accepted to evaluate the study. As you will see, the referees find the topic of your study of potential interest and are generally supportive. They raise, however, a series of concerns and make suggestions for modifications, which we would ask you to carefully address in a revision of the present work.

Please resubmit your revised manuscript online, with a covering letter listing amendments and responses to each point raised by the referees. Please resubmit the paper **within one month** and ideally as soon as possible. If we do not receive the revised manuscript within this time period, the file might be closed and any subsequent resubmission would be treated as a new manuscript. Please use the Manuscript Number (above) in all correspondence.

Additionally, I also include a list of more technical comments from our editorial assistance team. Please note that these must be also thoroughly corrected, but you do not need to address them in your point-by-point response letter.

Use the following link to submit your revised paper:

https://msb.msubmit.net/cgi-bin/main.plex

As a matter of course, please make sure that you have correctly followed the instructions for authors as given on the submission website.

Thank you for submitting this paper to Molecular Systems Biology.

Yours sincerely,

Yehu Moran
Academic Editor
Molecular Systems Biology

Please click on the link below to submit the revision online before 3rd May 2026.

https://msb.msubmit.net/cgi-bin/main.plex

We realize that it is difficult to revise to a specific deadline. In the interest of protecting the conceptual advance provided by the work, we recommend a revision within 3 months (3rd May 2026). Please discuss the revision progress ahead of this time with the editor if you require more time to complete the revisions. Use the link below to submit your revision:

https://msb.msubmit.net/cgi-bin/main.plex

IMPORTANT: Please read our guidelines for revised submissions: https://link.springer.com/journal/44320/submission-guidelines#cms-Revised-submissions

When you send your revision, we will require the following items:

1. the manuscript text in LaTeX, RTF or MS Word format
2. a letter with a detailed description of the changes made in response to the referees. Please specify clearly the exact places in the text (pages and paragraphs) where each change has been made in response to each specific comment given
3. three to four 'bullet points' highlighting the main findings of your study

4. a short 'blurb' text summarizing in two sentences the study (max. 250 characters)
5. a 'synopsis image' (550px width and 300px height, Illustrator, PowerPoint or jpeg format), which can be used as 'visual title' for the synopsis section of your paper.
6. Please include an author contributions statement after the Acknowledgements section
7. Please complete the Author Checklist, which will be published alongside the paper as part of EMBO's transparent process.
8. When assembling figures, please refer to our figure preparation guideline in order to ensure proper formatting and readability in print as well as on screen:
https://media.springernature.com/original/springer-cms/rest/v1/content/27825798/data/v1
9. Please note that corresponding authors are required to supply an ORCID ID for their name upon submission of a revised manuscript.
10. At EMBO Press we ask authors to provide source data for the main manuscript figures. You will receive a separate email with instructions for providing source data with your revised manuscript, including how to upload and organize the files.
11. Include a Reagents and Tools Table as part of the Methods section, which can be downloaded from our author guidelines.

*** PLEASE NOTE *** As part of the EMBO Press transparent editorial process initiative (see our Editorial at https://dx.doi.org/10.1038/msb.2010.72 , Molecular Systems Biology will publish online a Review Process File to accompany accepted manuscripts. When preparing your letter of response, please be aware that in the event of acceptance, your cover letter/point-by-point document will be included as part of this File, which will be available to the scientific community. More information about this initiative is available in our Instructions to Authors. If you have any questions about this initiative, please contact the editorial office (msb@embo.org).
----

specific comments by editorial assistance team

AFFILIATIONS: full details on institution should be included (e.g. city, country)
Author contributions/CRediT: Should be removed from manuscript text and provided only via the system.
FUNDING INFO: the funding in Acknowledgments needs to match the separate entries in the system - grants included in system need to be included in manuscript text as well.
FIGURES: Figure EV3 has two parts - needs to be either combined into one EV figure or moved to Appendix.
FIGURE CALLOUTS: missing for Figure 6A-C; callout for Supplementary Figure EV1 to be adjusted to Figure EV1; callout for Dataset EV4 to be renamed as such instead of Supplementary Dataset EV4
DATASET EV LEGENDS: Legends for Datasets should be removed from the manuscript text and included in each file as a separate sheet.
COMPUTER CODES: page 12
COMPUTER CODES README FILES: not provided
SYNOPSIS IMAGE: provided but needs to be in jpeg, TIFF or png format: must be 550 pixels wide x 200-600 pixels high
SOURCE DATA: provided; SD files need to be provided as one zip folder per figure each folder having clearly labeled panels.

Extra notes:
- Materials and Methods should be labeled as 'Methods'
- Acknowledgments to be placed after DA section
- Please note that the specific URL for PRJNA1153801 dataset is not provided in the data availability statement. should be fixed.

Figure Legends - Comments
- Please indicate the statistical test used for data analysis in the legends of figures 3, EV4.
- Please note that information related to n is missing in the legend of figure EV1. Please provide.
- Please note that the error bars are not defined in the legend of figure EV1. Please define.

specific reviewer comments

Reviewer #1:

The authors have only partially responded to my comments. Rather than providing an exhaustive list of the points that remain partially addressed, I would like to emphasize one issue that I consider critical for ensuring the validity of both the results and the conclusions presented in the manuscript. Specifically, the authors should experimentally demonstrate the independence of the two LOH systems. As noted previously, if the occurrence of one sLOH increases the probability of a second sLOH event, then the simulations used to estimate dLOH frequencies would be flawed. This would have significant implications for the interpretation of the results, particularly regarding the reported excess of dLOH events.

Reviewer #2:

Review of revised manuscript MSB-2025-13477R
"Burst of aneuploidy: A cost of adaptation driven by breakdown of cell cycle control"
Pirog, Tutaj, Tomala, Korona - Second review

The authors have responded constructively to the concerns raised in the first round of review and the manuscript is substantially improved. The addition of Figure 6 greatly aids comprehension of the experimental design. The deposition of the simulation code on GitHub, the comparison of three independent mutation rate estimation methods, and Dataset EV4 collectively address earlier concerns about simulation transparency. The clarification of the mutation accumulation structure, the median labeling in Figure 3, the worked dia2 example for Figure 4, the fitness cost simulations in Dataset EV5, and the tightened Discussion are all welcome improvements.

The remaining concern is that the Gene Ontology (GO) categories enrichment should only be performed against the preselected set of genes in the study. While an enrichment analysis is now presented in EV6, it uses the complete S. cerevisiae genome as the background. The README in EV6 acknowledges this issue, noting that the cell cycle checkpoint term remains enriched when the reference group is restricted to the ~400 starting strains - but this corrected analysis is not presented in the manuscript text or Dataset EV6 itself.

An enrichment test run against all yeast ORFs as background is inappropriate here because the 392-gene starting set was assembled by pre-selecting genes already known to increase genome instability, and is already heavily enriched for DNA repair, chromosome segregation, and cell cycle checkpoint functions relative to the genome as a whole. The only appropriate background is the 392-gene starting set itself. To illustrate the impact: a Fisher's exact test for GO:0000075 ('cell cycle checkpoint') against the whole-genome background yields $p \approx 7.9 \times 10^{-19}$, whereas the same test using the 392-gene starting set as background yields $p \approx 2.0 \times 10^{-7}$ - a difference of twelve orders of magnitude. Notably, the enrichment remains significant after background correction (11/39 hit genes vs. 20/392 background genes; 5.5-fold; FDR-adjusted $p \approx 1.3 \times 10^{-6}$), so the biological conclusion is likely to survive a properly conducted analysis.

The authors should resolve this in one of two ways:

1. Replace the enrichment analysis in Dataset EV6 with an analysis using the 392-gene starting set as the reference set and add a sentence to the Methods describing the test, background, and multiple-testing correction applied.

2. Remove the enrichment framing entirely and replace "enriched" with descriptive language (e.g., "represented" or "commonly annotated"). In this case, the 1.3% whole-genome frequency of GO:0000075 could be retained as contextual information rather than as a formal test statistic.

Reviewer #1:

The authors have only partially responded to my comments. Rather than providing an exhaustive list of the points that remain partially addressed, …

<<AUTHORS: Our reply to the questions raised by the reviewer was seven pages long as we wanted to address each of the many points raised by her or him. We have rewritten extensive portions of the former msc. and added several new graphs, datasets, statistical tests and simulations.>>

… I would like to emphasize one issue that I consider critical for ensuring the validity of both the results and the conclusions presented in the manuscript. Specifically, the authors should experimentally demonstrate the independence of the two LOH systems. As noted previously, if the occurrence of one sLOH increases the probability of a second sLOH event, then the simulations used to estimate dLOH frequencies would be flawed. This would have significant implications for the interpretation of the results, particularly regarding the reported excess of dLOH events.

<<AUTHORS: The mentioned "… independence of the two LOH systems" is a null hypothesis justified by the fact that the two loci reside on different chromosomes. It is not experimentally feasible to demonstrate it, in material/molecular sense. It is only possible to test for statistically significant deviations from it. The reviewer is apparently concerned with the hypothetical case that the first single LOH may occasionally make the second one more likely. This is conceivable, we admit it. However, we consider it fully legitimate to postulate that the second LOH is not affected by the first one, calculate the expected frequencies of double LOH and test whether empirical ones excess them. Transient hypermutability could be one of "real" explanations.>>

Reviewer #2:

Review of revised manuscript MSB-2025-13477R
"Burst of aneuploidy: A cost of adaptation driven by breakdown of cell cycle control"
Pirog, Tutaj, Tomala, Korona - Second review

The authors have responded constructively to the concerns raised in the first round of review and the manuscript is substantially improved. The addition of Figure 6 greatly aids comprehension of the experimental design. The deposition of the simulation code on GitHub, the comparison of three independent mutation rate estimation methods, and Dataset EV4 collectively address earlier concerns about simulation transparency. The clarification of the mutation accumulation structure, the median labeling in Figure 3, the worked dia2 example for Figure 4, the fitness cost simulations in Dataset EV5, and the tightened Discussion are all welcome improvements.

<<AUTHORS: We are pleased to hear it.>>

The remaining concern is that the Gene Ontology (GO) categories enrichment should only be performed against the preselected set of genes in the study. While an enrichment analysis is now

presented in EV6, it uses the complete S. cerevisiae genome as the background. The README in EV6 acknowledges this issue, noting that the cell cycle checkpoint term remains enriched when the reference group is restricted to the ~400 starting strains - but this corrected analysis is not presented in the manuscript text or Dataset EV6 itself.

An enrichment test run against all yeast ORFs as background is inappropriate here because the 392-gene starting set was assembled by pre-selecting genes already known to increase genome instability, and is already heavily enriched for DNA repair, chromosome segregation, and cell cycle checkpoint functions relative to the genome as a whole. The only appropriate background is the 392-gene starting set itself. To illustrate the impact: a Fisher's exact test for GO:0000075 ('cell cycle checkpoint') against the whole-genome background yields $p \approx 7.9 \times 10^{-19}$, whereas the same test using the 392-gene starting set as background yields $p \approx 2.0 \times 10^{-7}$ - a difference of twelve orders of magnitude. Notably, the enrichment remains significant after background correction (11/39 hit genes vs. 20/392 background genes; 5.5-fold; FDR-adjusted $p \approx 1.3 \times 10^{-6}$), so the biological conclusion is likely to survive a properly conducted analysis.

The authors should resolve this in one of two ways:

1. Replace the enrichment analysis in Dataset EV6 with an analysis using the 392-gene starting set as the reference set and add a sentence to the Methods describing the test, background, and multiple-testing correction applied.

2. Remove the enrichment framing entirely and replace "enriched" with descriptive language (e.g., "represented" or "commonly annotated"). In this case, the 1.3% whole-genome frequency of GO:0000075 could be retained as contextual information rather than as a formal test statistic.

<<AUTHORS: In the first revision, we were trying to say that all previous studies on LOH involved all yeast genes and tended to identify those involved in cell cycle checkpoints as being extremely overrepresented. Therefore, we compared our strains with the highest dLOH frequencies with the entire yeast gene set. The reviewer suggested leaving only the tests involving the genes we studied and referring to comparisons with the whole genome as 'contextual information'. We are grateful for this thoughtful advice and accept points 1 and 2 (there is no need to choose between them). The small changes introduced to the text can be found in lines 201–203.

30th Apr 2026
Manuscript number: MSB-2025-13477RR
Title: Burst of aneuploidy: A cost of adaptation driven by breakdown of cell cycle control

Dear Dr Korona,

Thank you again for sending us your revised manuscript. We are now satisfied with the modifications made and I am pleased to inform you that your paper has been accepted for publication.

Importantly, please note that we would not be able to send your paper for production before you provide us with a synopsis image following EXACTLY these criteria: TIFF or png format: 550 pixels width and 300 pixels height. Please send us this file at your earliest convenience via email to avoid unnecessary delays with your manuscript.

Your manuscript will be processed for publication by EMBO Press. It will be copy edited and you will receive page proofs prior to publication. Please note that you will be contacted by Springer Nature Author Services to complete licensing and payment information.

You may qualify for financial assistance for your publication charges - either via a Springer Nature fully open access agreement or an EMBO initiative. Check your eligibility: https://link.springer.com/journal/44320/how-to-publish-with-us

Should you be planning a Press Release on your article, please get in contact with embo_production@springernature.com as early as possible in order to coordinate publication and release dates.

If you have any questions, please do not hesitate to contact the Editorial Office. Thank you for your contribution to Molecular Systems Biology.

Yours sincerely,

Sincerely,

Yehu Moran
Editor
Molecular Systems Biology

>>> Please note that it is Molecular Systems Biology policy for the transcript of the editorial process (containing referee reports and your response letter) to be published as an online supplement to each paper. If you do NOT want this, you will need to inform the Editorial Office via email immediately. More information is available here: https://link.springer.com/partners/embo-press/editorial-policies#Peer%20review

EMBO Press Author Checklist

Corresponding Author Name: Adrian Pirog, Ryszard Korona	
Journal Submitted to: Molecular Systems Biology	
Manuscript Number: MSB-2025-13477	

USEFUL LINKS FOR COMPLETING THIS FORM
The EMBO Journal - Author Guidelines
EMBO Reports - Author Guidelines
Molecular Systems Biology - Author Guidelines
EMBO Molecular Medicine - Author Guidelines

Reporting Checklist for Life Science Articles (updated January
This checklist is adapted from Materials Design Analysis Reporting (MDAR) Checklist for Authors. MDAR establishes a minimum set of requirements in transparent reporting in the life sciences (see Statement of Task: 10.31222/osf.io/9sm4x). Please follow the journal's guidelines in preparing your
Please note that a copy of this checklist will be published alongside your article.

Abridged guidelines for figures
1. Data
The data shown in figures should satisfy the following conditions:
- the data were obtained and processed according to the field's best practice and are presented to reflect the results of the experiments in an accurate and unbiased manner.
- ideally, figure panels should include only measurements that are directly comparable to each other and obtained with the same assay.
- plots include clearly labeled error bars for independent experiments and sample sizes. Unless justified, error bars should not be shown for technical
- if n<5, the individual data points from each experiment should be plotted. Any statistical test employed should be justified.
- Source Data should be included to report the data underlying figures according to the guidelines set out in the authorship guidelines on Data

2. Captions
Each figure caption should contain the following information, for each panel where they are relevant:
- a specification of the experimental system investigated (eg cell line, species name).
- the assay(s) and method(s) used to carry out the reported observations and measurements.
- an explicit mention of the biological and chemical entity(ies) that are being measured.
- an explicit mention of the biological and chemical entity(ies) that are altered/varied/perturbed in a controlled manner.
- the exact sample size (n) for each experimental group/condition, given as a number, not a range;
- a description of the sample collection allowing the reader to understand whether the samples represent technical or biological replicates (including how many animals, litters, cultures, etc.).
- a statement of how many times the experiment shown was independently replicated in the laboratory.
- definitions of statistical methods and measures:
  - common tests, such as t-test (please specify whether paired vs. unpaired), simple χ2 tests, Wilcoxon and Mann-Whitney tests, can be unambiguously identified by name only, but more complex techniques should be described in the methods section;
  - are tests one-sided or two-sided?
  - are there adjustments for multiple comparisons?
  - exact statistical test results, e.g., P values = x but not P values < x;
  - definition of 'center values' as median or average;
  - definition of error bars as s.d. or s.e.m.

Please complete ALL of the questions below.
Select "Not Applicable" only when the requested information is not relevant for your study.

Materials

Newly Created Materials	Information included in the manuscript?	In which section is the information available? (Reagents and Tools Table, Materials and Methods, Figures, Data Availability Section)
New materials and reagents need to be available; do any restrictions apply?	Not Applicable	

Antibodies	Information included in the manuscript?	In which section is the information available? (Reagents and Tools Table, Materials and Methods, Figures, Data Availability Section)
For **antibodies** provide the following information:<br>- Commercial antibodies: RRID (if possible) or supplier name, catalogue number and or/clone number<br>- Non-commercial: RRID or citation	Not Applicable	

DNA and RNA sequences	Information included in the manuscript?	In which section is the information available? (Reagents and Tools Table, Materials and Methods, Figures, Data Availability Section)
**Short novel DNA or RNA including primers, probes**: provide the sequences.	Not Applicable	

Cell materials	Information included in the manuscript?	In which section is the information available? (Reagents and Tools Table, Materials and Methods, Figures, Data Availability Section)
**Cell lines:** Provide species information, strain. Provide accession number in repository **OR** supplier name, catalog number, clone number, and/**OR** RRID.	Not Applicable	
**Primary cultures:** Provide species, strain, sex of origin, genetic modification status.	Not Applicable	
Report if the cell lines were recently **authenticated** (e.g., by STR profiling) and tested for mycoplasma contamination.	Not Applicable	

Experimental animals	Information included in the manuscript?	In which section is the information available? (Reagents and Tools Table, Materials and Methods, Figures, Data Availability Section)
**Laboratory animals or Model organisms:** Provide species, strain, sex, age, genetic modification status. Provide accession number in repository **OR** supplier name, catalog number, clone number, **OR** RRID.	Not Applicable	
**Animal observed in or captured from the field:** Provide species, sex, and age where possible.	Not Applicable	
Please detail **housing and husbandry conditions**.	Not Applicable	

Plants and microbes	Information included in the manuscript?	In which section is the information available? (Reagents and Tools Table, Materials and Methods, Figures, Data Availability Section)
**Plants:** provide species and strain, ecotype and cultivar where relevant, unique accession number if available, and source (including location for collected wild specimens).	Not Applicable	
**Microbes:** provide species and strain, unique accession number if available, and source.	Yes	Materials and Methods

Human research participants	Information included in the manuscript?	In which section is the information available? (Reagents and Tools Table, Materials and Methods, Figures, Data Availability Section)
If collected and within the bounds of privacy constraints report on age, sex and gender or ethnicity for all study participants.	Not Applicable	

Core facilities	Information included in the manuscript?	In which section is the information available? (Reagents and Tools Table, Materials and Methods, Figures, Data Availability Section)
If your work benefited from core facilities, was their service mentioned in the acknowledgments section?	Not Applicable	

Design

Study protocol	Information included in the manuscript?	In which section is the information available? (Reagents and Tools Table, Materials and Methods, Figures, Data Availability Section)
If study protocol has been **pre-registered, provide DOI in the manuscript**. For clinical trials, provide the trial registration number **OR** cite DOI.	Not Applicable	
Report the **clinical trial registration number** (at ClinicalTrials.gov or equivalent), where applicable.	Not Applicable	

Laboratory protocol	Information included in the manuscript?	In which section is the information available? (Reagents and Tools Table, Materials and Methods, Figures, Data Availability Section)
Provide DOI OR other citation details if **external detailed step-by-step protocols** are available.	Not Applicable	

Experimental study design and statistics	Information included in the manuscript?	In which section is the information available? (Reagents and Tools Table, Materials and Methods, Figures, Data Availability Section)
Include a statement about **sample size** estimate even if no statistical methods were used.	Yes	Materials and Methods
Were any steps taken to minimize the effects of subjective bias when allocating animals/samples to treatment (e.g. **randomization procedure**)? If yes, have they been described?	Yes	Materials and Methods
Include a statement about **blinding** even if no blinding was done.	Yes	Materials and Methods
Describe **inclusion/exclusion criteria** if samples or animals were excluded from the analysis. Were the criteria pre-established?<br><br>If sample or data points were omitted from analysis, report if this was due to attrition or intentional exclusion and provide justification.	Yes	Materials and Methods
For every figure, are **statistical tests** justified as appropriate? Do the data meet the assumptions of the tests (e.g., normal distribution)? Describe any methods used to assess it. Is there an estimate of variation within each group of data? Is the variance similar between the groups that are being statistically compared?	Yes	Materials and Methods

Sample definition and in-laboratory replication	Information included in the manuscript?	In which section is the information available? (Reagents and Tools Table, Materials and Methods, Figures, Data Availability Section)
In the figure legends: state number of times the experiment was **replicated** in laboratory.	Yes	Materials and Methods
In the figure legends: define whether data describe **technical or biological replicates**.	Yes	Materials and Methods

Ethics

Ethics	Information included in the manuscript?	In which section is the information available? (Reagents and Tools Table, Materials and Methods, Figures, Data Availability Section)
Studies involving **human participants**: State details of **authority granting ethics approval** (IRB or equivalent committee(s), provide reference number for approval.	Not Applicable	
Studies involving **human participants**: Include a statement confirming that **informed consent** was obtained from all subjects and that the experiments conformed to the principles set out in the WMA Declaration of Helsinki and the Department of Health and Human Services Belmont Report.	Not Applicable	
Studies involving **human participants:** For publication of **patient photos**, include a statement confirming that consent to publish was obtained.	Not Applicable	
Studies involving experimental **animals**: State details of **authority granting ethics approval** (IRB or equivalent committee(s), provide reference number for approval. Include a statement of compliance with ethical regulations.	Not Applicable	
Studies involving **specimen and field samples:** State if relevant **permits** obtained, provide details of authority approving study; if none were required, explain why.	Not Applicable	

Dual Use Research of Concern (DURC)	Information included in the manuscript?	In which section is the information available? (Reagents and Tools Table, Materials and Methods, Figures, Data Availability Section)
Could your study fall under dual use research restrictions? Please check biosecurity documents and list of **select agents and toxins** (CDC): https://www.selectagents.gov/sat/list.htm	Not Applicable	
If you used a select agent, is the security level of the lab appropriate and reported in the manuscript?	Not Applicable	
If a study is subject to dual use research of concern regulations, is the name of the **authority granting approval and reference number** for the regulatory approval provided in the manuscript?	Not Applicable	

Reporting

The MDAR framework recommends adoption of discipline-specific guidelines, established and endorsed through community initiatives. Journals have their own policy about requiring specific guidelines and recommendations to complement MDAR.

Adherence to community standards	Information included in the manuscript?	In which section is the information available? (Reagents and Tools Table, Materials and Methods, Figures, Data Availability Section)
State if relevant guidelines or checklists (e.g., **ICMJE, MIBBI, ARRIVE, PRISMA**) have been followed or provided.	Not Applicable	
For **tumor marker prognostic studies,** we recommend that you follow the **REMARK** reporting guidelines (see link list at top right). See author guidelines, under 'Reporting Guidelines'. Please confirm you have followed these guidelines.	Not Applicable	
For **phase II and III randomized controlled trials**, please refer to the **CONSORT** flow diagram (see link list at top right) and submit the CONSORT checklist (see link list at top right) with your submission. See author guidelines, under 'Reporting Guidelines'. Please confirm you have submitted this list.	Not Applicable	

Data Availability

Data availability	Information included in the manuscript?	In which section is the information available? (Reagents and Tools Table, Materials and Methods, Figures, Data Availability Section)
Have **primary datasets** been deposited according to the journal's guidelines (see 'Data Deposition' section) and the respective accession numbers provided in the Data Availability Section?	Yes	Data Availability Section
Were **human clinical and genomic datasets** deposited in a public access-controlled repository in accordance to ethical obligations to the patients and to the applicable consent agreement?	Not Applicable	
Are **computational models** that are central and integral to a study available without restrictions in a machine-readable form? Were the relevant accession numbers or links provided?	Yes	Materials and Methods
If publicly available data were reused, provide the respective **data citations in the reference list**.	Not Applicable	
